# Predictive Value of Early Autism Detection Models Based on Electronic Health Record Data Collected Before Age 1 Year

**DOI:** 10.1001/jamanetworkopen.2022.54303

**Published:** 2023-02-02

**Authors:** Matthew M. Engelhard, Ricardo Henao, Samuel I. Berchuck, Junya Chen, Brian Eichner, Darby Herkert, Scott H. Kollins, Andrew Olson, Eliana M. Perrin, Ursula Rogers, Connor Sullivan, YiQin Zhu, Guillermo Sapiro, Geraldine Dawson

**Affiliations:** 1 Department of Biostatistics and Bioinformatics, Duke University School of Medicine, Durham, North Carolina; 2 Department of Electrical and Computer Engineering, Duke University, Durham, North Carolina; 3 Duke AI Health, Durham, North Carolina; 4 Department of Statistical Science, Duke University, Durham, North Carolina; 5 Department of Pediatrics, Duke University School of Medicine, Durham, North Carolina; 6 Department of Psychiatry and Behavioral Sciences, Duke University School of Medicine, Durham, North Carolina; 7 Department of Pediatrics, Johns Hopkins University School of Medicine, Baltimore, Maryland; 8 Department of Pediatrics, Johns Hopkins University School of Nursing, Baltimore, Maryland; 9 Duke Institute for Brain Sciences, Durham, North Carolina

## Abstract

**Importance:**

Autism detection early in childhood is critical to ensure that autistic children and their families have access to early behavioral support. Early correlates of autism documented in electronic health records (EHRs) during routine care could allow passive, predictive model-based monitoring to improve the accuracy of early detection.

**Objective:**

To quantify the predictive value of early autism detection models based on EHR data collected before age 1 year.

**Design, Setting, and Participants:**

This retrospective diagnostic study used EHR data from children seen within the Duke University Health System before age 30 days between January 2006 and December 2020. These data were used to train and evaluate L2-regularized Cox proportional hazards models predicting later autism diagnosis based on data collected from birth up to the time of prediction (ages 30-360 days). Statistical analyses were performed between August 1, 2020, and April 1, 2022.

**Main Outcomes and Measures:**

Prediction performance was quantified in terms of sensitivity, specificity, and positive predictive value (PPV) at clinically relevant model operating thresholds.

**Results:**

Data from 45 080 children, including 924 (1.5%) meeting autism criteria, were included in this study. Model-based autism detection at age 30 days achieved 45.5% sensitivity and 23.0% PPV at 90.0% specificity. Detection by age 360 days achieved 59.8% sensitivity and 17.6% PPV at 81.5% specificity and 38.8% sensitivity and 31.0% PPV at 94.3% specificity.

**Conclusions and Relevance:**

In this diagnostic study of an autism screening test, EHR-based autism detection achieved clinically meaningful accuracy by age 30 days, improving by age 1 year. This automated approach could be integrated with caregiver surveys to improve the accuracy of early autism screening.

## Key Points

**Question:**

Can autism be detected from routine electronic health records (EHRs) with clinically meaningful accuracy before age 1 year?

**Findings:**

In this diagnostic study of 45 080 children, the accuracy of EHR-based early autism detection models at age 30 days was competitive with caregiver surveys collected at ages 18 to 24 months. Model accuracy improved further by age 1 year.

**Meaning:**

These findings suggest that EHR-based autism detection could be integrated with caregiver surveys to improve the accuracy of early autism screening.

## Teaser

This diagnostic study evaluates the accuracy of early autism detection models based on electronic health record data collected before age 1 year.

## Introduction

Detection of autism early in childhood is critical to ensure that autistic children and their families have access to appropriate supportive resources. In particular, early detection is a necessary step toward early behavioral support, which has been associated with improved outcomes.^1,2,3^ To improve rates of early detection, the American Academy of Pediatrics has recommended universal screening at age 18 to 24 months,^4,5^ and a recent Lancet Commission on the future of care and clinical research in autism reaffirmed the importance of prompt access to supportive services to help autistic children develop and succeed.^6^ However, results from the US Autism and Developmental Disabilities Monitoring Network^7^ showed that in 2018, the median age at first diagnosis was 50 months, implying that most autistic children are still identified too late to fully benefit from early support.

The most commonly used early autism screening tools are the Modified Checklist for Autism in Toddlers With Follow-up (M-CHAT-F) and its revised version (M-CHAT-R/F), which are valid from age 16 to 30 months. Recent analysis in a large pediatric network found that the M-CHAT-F detects autism with 39% sensitivity and 15% positive predictive value (PPV).^8^ Another early screening measure, the Social Attention and Communication Surveillance–Revised, outperformed the M-CHAT-F, reaching 62% sensitivity and 83% PPV between ages 12 and 24 months.^9^ A third measure, the Parent’s Observations of Social Interaction, showed 83% sensitivity at 75% specificity in a combined primary care and subspecialty sample of 232 children.^10^ While these measures are essential tools supporting early detection, there is still a need to develop new approaches and use additional sources of information to boost their accuracy and reliability. Furthermore, alternative approaches may be better suited to mitigate subjectivity and biases in existing measures. For example, the M-CHAT-F performs worse among girls, racial and ethnic minority children, and children from lower-income households.^8^ These biases may contribute to disparities in diagnosis, such as the delays in diagnosis observed in girls^11^ and the delays and lower rates of diagnosis observed in racial and ethnic minority indiviuals.^12,13^ Finally, the aforementioned measures are recommended at age 16 to 30 months, but earlier suspicion may improve oversight or facilitate more timely support.

Passive monitoring of electronic health record (EHR) data is a promising alternative approach to early detection. A variety of known early correlates of autism are documented in the EHR, including low birth weight,^14^ preterm birth, low Apgar scores, and other perinatal complications.^15,16^ Early autism-related conditions,^17^ such as postnatal hyperbilirubinemia^18^ and respiratory infections,^19^ are also documented using diagnosis codes. In addition, problems with crying, sleeping, and feeding, which are associated with later autism diagnosis,^20^ may be documented in clinical notes or reflected in high rates of visits to specific health services. Any of these findings has limited predictive value in isolation, but collectively, EHR data may be adequate to detect autism effectively from a very early age. In our previous work, we found that autism was associated with distinctive patterns of health care use before age 1 year, such as increased rates of intubation and ventilation, physical therapy visits, and ophthalmology visits.^21^ More recently, Onishchenko et al^22^ demonstrated that predictive models developed using claims data—and based only on the patient’s previous diagnosis codes—provided meaningful information about autism as early as age 50 weeks and were sufficient to detect autism with 52% sensitivity and 16% to 19% PPV (for male and female individuals, respectively) at age 150 weeks. As these results showed, the performance of these models was superior to the M-CHAT. Furthermore, similar performance was observed when the models were applied to diagnosis records from the University of Chicago Medical Center.^22^

We hypothesized that models incorporating a more comprehensive set of EHR-based predictors in addition to diagnosis codes could detect autism with performance exceeding that of the M-CHAT much earlier than 29 months, the age required to reach this level of performance using diagnostic codes alone.^22^ Based on our previous observation that distinctive patterns of health care use begin at a very early age as well as the large number of autism-related findings that occur in the perinatal period, we further hypothesized that autism detection would be possible much earlier than age 1 year and as early as 30 days after birth. To investigate these hypotheses, we leveraged more than 14 years of EHR data from the Duke University Health System (DUHS), a large academic medical center located in and around Durham, North Carolina, to train and evaluate EHR-based early autism detection models.

Our evaluation focused primarily on the association of patient age at the time of prediction (ie, the data collection window) with autism detection performance. However, we also aimed to thoroughly explore differences in performance between groups defined by sex, race and ethnicity, and other demographic variables as a preliminary exploration of potential biases associated with EHR-based autism detection. Last, we aimed to quantify the effect of other neurodevelopmental conditions, including attention-deficit/hyperactivity disorder (ADHD), on autism detection performance. More than 40% of autistic children have co-occurring ADHD symptoms,^23,24^ and their quality of life^25^ and adaptive functioning are lower compared with autistic children without co-occurring ADHD.^25,26^ Early identification is particularly challenging in this group because autism diagnosis is delayed by a mean of 3 years in those first diagnosed with ADHD^27^; therefore, it is important to assess whether EHR-based detection is effective in these groups specifically.

## Methods

The procedures used in this diagnostic study were approved by the Duke Health Institutional Review Board (eMethods in Supplement 1). Participant consent was waived due to the minimal risk posed by the study procedures and infeasibility of obtaining consent in a large retrospective cohort. The study followed the Transparent Reporting of a Multivariable Prediction Model for Individual Prognosis or Diagnosis (TRIPOD) reporting guideline for prediction model development and validation.

### Data Source

All results were based on retrospective data analysis of EHRs from DUHS. This health system provides care to approximately 85% of children in Durham and surrounding Durham County, which has a diverse, majority racial and ethnic minority population with varying demographic and socioeconomic status.^28^ Records were extracted from the current DUHS EHR (2013 to present), which is based on the platform developed by Epic, as well as several EHR platforms operating before 2013. This study was conducted at the Duke University School of Medicine between August 1, 2020, and April 1, 2022. All analyses were executed within the Duke Protected Analytics Computing Environment, a highly protected virtual network space designed for protected health information.

Study inclusion criteria were as follows: (1) date of birth between October 1, 2006, and December 1, 2019; (2) at least 1 recorded encounter within the DUHS before age 30 days (between January 2006 and December 2020); and (3) at least 2 total recorded encounters within the DUHS before age 1 year. Criteria 1 and 2 were designed to ensure that model input features were available before age 30 days and that similar criteria could be applied in a prospective model evaluation or deployment. Data associated with DUHS encounters occurring between October 1, 2006, and June 1, 2021, were extracted for patients meeting the inclusion criteria. Demographic information, including race and ethnicity, was determined based on available EHR fields.

### Case Definitions and Cohort Selection

Autism (autism spectrum disorder) and other neurodevelopmental conditions were identified using computable phenotypes based on billing codes from the *International Classification of Diseases, Ninth Revision, Clinical Modification* (*ICD-9-CM*) and the *International Classification of Diseases, Tenth Revision, Clinical Modification* (*ICD-10-CM*). For a particular condition, a patient was defined as a case if (1) codes for that condition were documented on 2 or more distinct calendar dates or (2) a code for that condition was associated with an encounter at a DUHS clinic specializing in neurodevelopmental disorders. The *ICD-9-CM* codes 299.00, 299.01, 299.80, 299.81, and 299.90^29^ as well as *ICD-10-CM* codes F84.0, F84.5, F84.8, and F84.9 were associated with autism. A complete list of codes associated with ADHD, intellectual disability, genetic neurodevelopmental conditions, and other neurodevelopmental conditions can be found in eTable 3 in Supplement 1.

Patients meeting criteria for any of these conditions were included in the analysis. In addition, a population of control participants not meeting criteria for any of these conditions was selected for inclusion. Since the prevalence of each condition increases with age, our selection of control participants was stratified by year of birth to avoid possible bias. Specifically, we selected as many control participants as possible while maintaining the same ratio of autism case patients to control participants across all birth years. This procedure was also designed to yield a sample-specific autism prevalence that was similar to the prevalence of autism within the DUHS overall.

### Model Development and Evaluation

Prediction models were based on EHRs recorded by ages 30, 60, 90, 180, 270, and 360 days. The date of the last observed DUHS encounter was taken as the date of last follow-up (ie, right-censoring time). Data were divided at random into a development set (60%) used to train models and tune hyperparameters and a test set (40%) used to evaluate the performance of the final model. An L2-regularized Cox proportional hazards model was selected following evaluation of multiple competing approaches. The area under the receiver operating characteristic curve (AUC), the average (mean) positive predictive value (AP), and the concordance index were used to evaluate performance. Since lifetime diagnosis status was highly uncertain for patients with a short follow-up, we primarily report the AUC_t_ and AP_t_, defined as the AUC and AP when limiting negative cases to individuals followed up for at least *t* years. Additional model development and evaluation details are presented in the eMethods in Supplement 1.

### Statistical Analysis

Differences in rates of each sex, racial and ethnic group, and other neurodevelopmental conditions between autism case patients and control participants were calculated by cross-tabulating each variable with autism diagnosis and applying a χ^2^ test. Differences between autism case patients and control participants in the number of encounters in the first year of life were compared with the Mann-Whitney test. The Nelson-Aalen estimator was used to estimate the cumulative rate of autism diagnosis between ages 0 and 14 years. Differences in discrimination performance between models were tested for statistical significance by applying a DeLong test to the respective AUC values.^30^ The average influence of each predictor (diagnosis codes, procedure codes, laboratory measurements, medications, vital signs, and encounter details) on model predictions (ie, feature importance) for each model was quantified by calculating the mean absolute value of all Shapley additive explanation values^31^ for that predictor on individuals in the test set. The influence of each predictor group (eg, diagnoses, demographics) was calculated by summing these values across all predictors in that group. Pearson correlations were calculated to quantify trends in the influence of each predictor group over time. Test statistics were assessed for statistical significance at a threshold of α = .05. Statistical analyses were conducted between August 1, 2020, and April 1, 2022, using scipy for Python version 3.7. Also, as mentioned in the eMethods in Supplement 1, model development was in scikit-survival for Python version 3.7.

## Results

### Description of Cohort

A total of 63 016 children met the study eligibility criteria (eResults in Supplement 1). Of these individuals, 924 (1.5%) satisfied our autism computable phenotype, with a median age of 5.2 years (IQR, 3.5-7.6 years) at the time of diagnosis. An additional 175 individuals had at least 1 autism-related diagnosis code but did not satisfy our autism diagnosis criteria. These individuals were included only in secondary analyses exploring sensitivity to our computable phenotype. Among those not meeting autism criteria, 10 782 met criteria for ADHD or another neurodevelopmental condition and were therefore included. A total of 47 540 individuals had no diagnosis codes related to a neurodevelopmental condition and were therefore eligible for selection as control participants. Of these, 33 374 were selected to balance the age of birth distribution between the autism and control groups. In total, 45 080 individuals were included in the study (eFigure 1 in Supplement 1). The number of encounters observed by age in autism case patients and control participants is summarized in eFigure 2 of Supplement 1, and the estimated prevalence of autism diagnosis by age is summarized in eFigure 3 of Supplement 1. The 924 case patients consisted of 738 males (79.1%), 186 females (20.1%), and 8 American Indian or Alaska Native (0.9%), 32 Asian (3.5%), 323 Black (35.0%), 3 Native Hawaiian or Pacific Islander (0.3%), 369 White (39.9%), and 40 multiracial (4.3%) children as well as 149 children (16.1%) whose race was unknown. Characteristics of all the study participants are summarized in eTable 1 in Supplement 1.

### Prediction Performance Over Time

Of the 45 080 individuals included in this study, 18 032 were randomly assigned to the test set, including 363 autism case patients and 3721 control participants meeting the computable phenotype for 1 or more other neurodevelopmental conditions. A total of 3615 control participants were followed up through age 8 years and were therefore included when calculating our primary performance measures (eg, AUC_8_).

**Figure 1.**
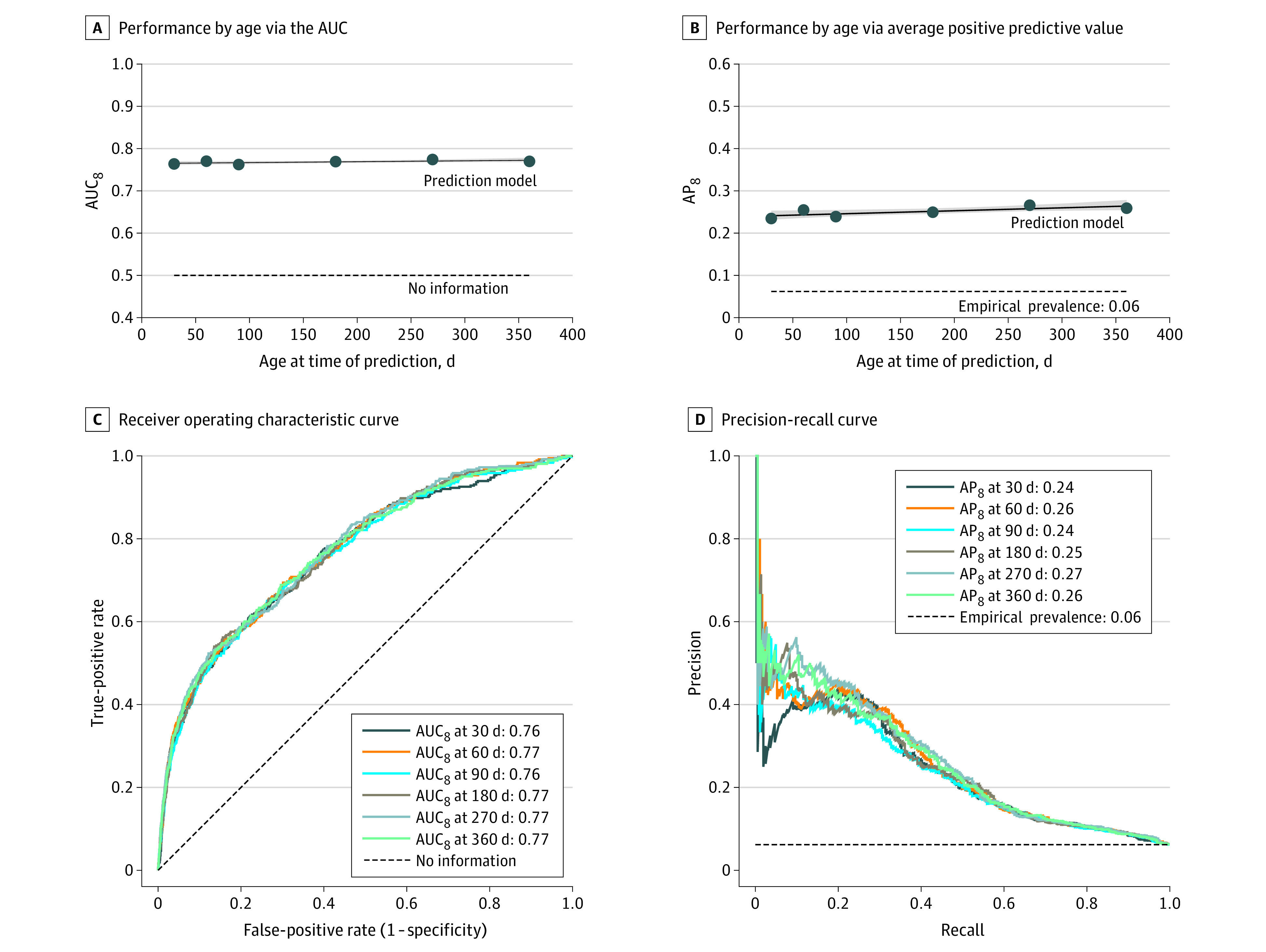
Autism Model Prediction Performance by Age at Time of Prediction Prediction performance is shown for models based on data collected from birth through ages 30, 60, 90, 180, 270, and 360 days, respectively. Case patients were defined as children later meeting our autism computable phenotype, and control participants were defined as children followed up through age 8 years but not meeting our phenotype. AP_8_ indicates average (mean) positive predictive value for individuals followed up through age 8 years; AUC_8_, area under the receiver operating characteristic curve for individuals followed up through age 8 years.

As shown in Figure 1, the AUC_8_ ranged from 0.76 at 90 days to 0.77 at 270 days. In contrast, the AP_8_ increased from 0.24 at 30 days, a 3.8-fold increase over the autism rate (6.2%), to 0.27 at 270 days, a 4.3-fold increase over the autism rate. When control participants with other neurodevelopmental conditions were excluded (eFigure 4 in Supplement 1), the AUC_8_ ranged from 0.79 at 30 days to 0.82 at 270 days, and the AP_8_ ranged from 0.41 at 30 days to 0.49 at 270 days. The sensitivity of these results to the required follow-up threshold *t* is summarized in eFigures 5 and 6 in Supplement 1. The concordance index ranged from 0.765 at 30 days to 0.774 at 360 days, as shown in eTable 2 in Supplement 1.

**Figure 2.**
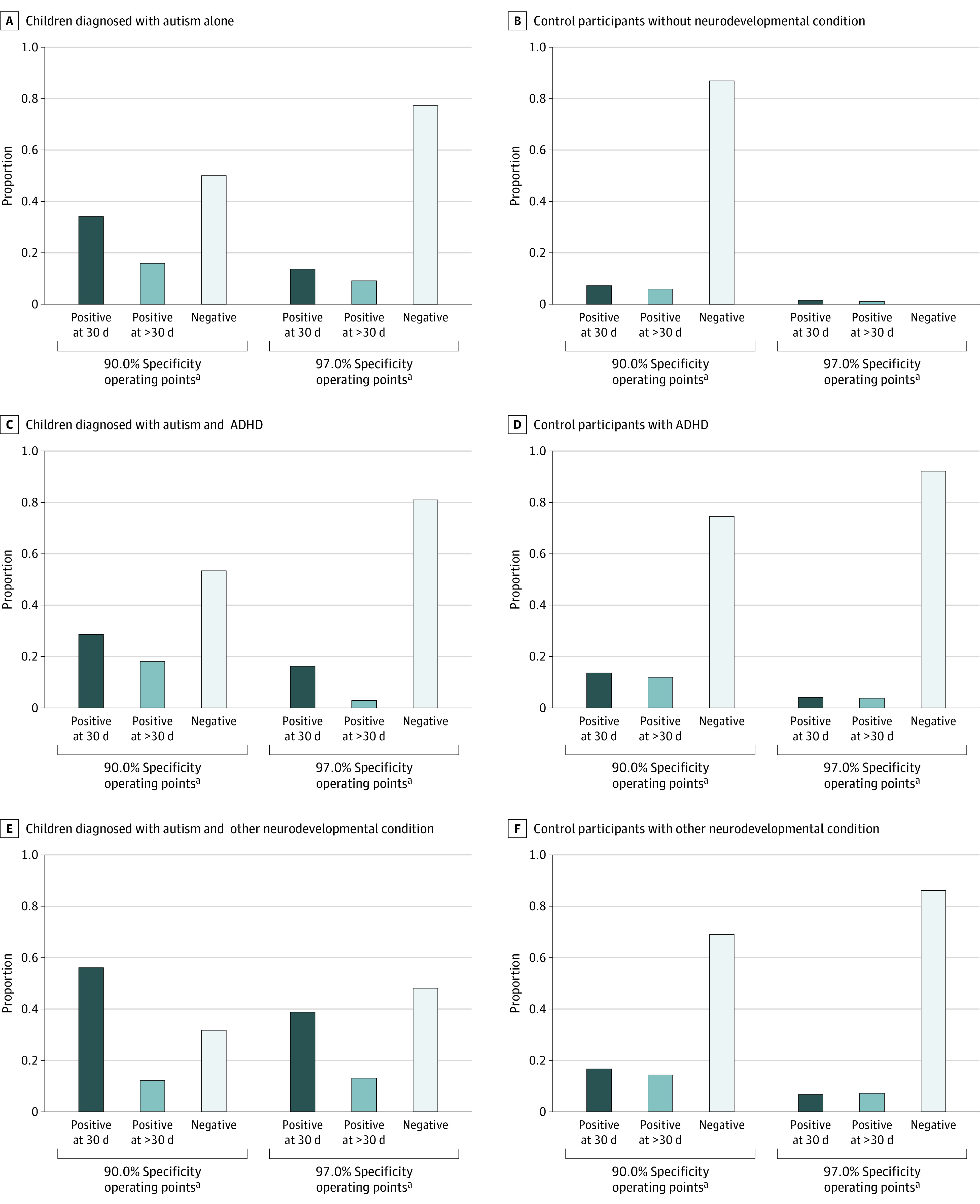
Identification of Autistic Children and Control Participants Stratified by Other Neurodevelopmental Conditions at Selected Operating Points Prediction performance is shown for individuals later diagnosed with attention-deficit/hyperactivity disorder (ADHD), another neurodevelopmental condition, or neither. In each of these groups, case patients were defined as children later meeting our autism computable phenotype, and control participants were defined as children followed up through age 8 years but not meeting our phenotype. ^a^Operating points were selected for each model (30, 60, 90, 180, 270, and 360 days) individually to achieve specificity greater than or equal to the stated value.

Model performance over time at several operating points with varying sensitivity and specificity is summarized in Figure 2 and in the eResults and eTable 2 in Supplement 1. The operating points are depicted in eFigure 7 in Supplement 1. Model-based autism detection at age 30 days achieved 45.5% sensitivity and 23.0% PPV at 90.0% specificity. Detection by 360 days achieved 59.8% sensitivity and 17.6% PPV at 81.5% specificity, and 38.8% sensitivity and 31.0% PPV at 94.3% specificity. Model calibration is summarized in eFigure 8 of Supplement 1.

### Prediction Among Individuals With Other Neurodevelopmental Conditions

**Figure 3.**
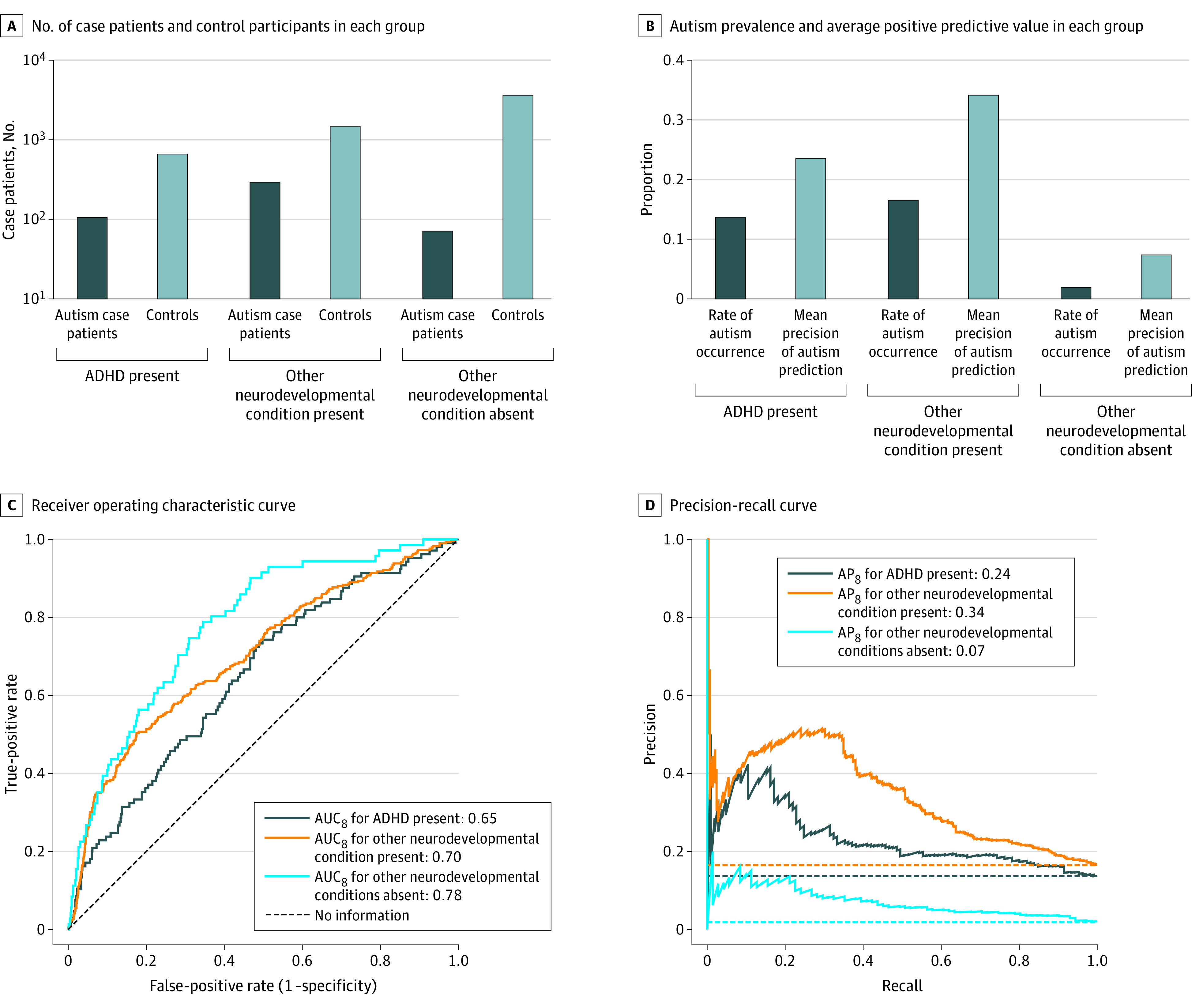
Autism Model Prediction Performance at 30 Days for Individuals With and Without Other Neurodevelopmental Conditions A, C, and E, Proportion of children later diagnosed with autism correctly identified at 30 days, after 30 days, and not identified by 360 days. B, D, and F, Proportion of false-positive results at 30 days and after 30 days among children not diagnosed at least through age 8 years. A and B are limited to children who did not later meet criteria for any other neurodevelopmental condition, whereas C through F include children later meeting criteria for attention-deficit/hyperactivity disorder (ADHD) and any other neurodevelopmental condition. Positive and negative predictions were based on the high-sensitivity (90%) and high-specificity (90%) operating points shown in eFigure 7 in Supplement 1.

We also evaluated the model’s ability to detect autism among individuals in the test set with other neurodevelopmental conditions. There were 768 individuals with an ADHD diagnosis and adequate follow-up; 105 (13.7%) also had an autism diagnosis. Limited to these 768 individuals, the AUC_8_ was 0.65 at 30 days (Figure 3) and 0.66 at 360 days (eFigure 9 in Supplement 1). The AP_8_ was 0.24 at 30 days and 0.24 at 360 days, a 1.7-fold increase over the autism rate. There were 1767 individuals with other neurodevelopmental conditions and adequate follow-up; 292 (16.5%) also had an autism diagnosis. Limited to these 1767 individuals, the AUC_8_ was 0.70 at 30 days and 0.69 at 360 days. The AP_8_ was 0.34 at 30 days and remained at 0.34 at 360 days, a 2.1-fold change over the autism rate. Corresponding results could not be obtained for genetic neurodevelopmental conditions and intellectual disability due to their low rates in the test set (n = 3 and 20, respectively).

Last, we evaluated the model’s ability to identify the 71 individuals with an autism diagnosis among the 3686 individuals with no other identified neurodevelopmental condition. Limited to these 3686 individuals, the AUC_8_ was 0.78 at 30 days and 0.77 at 360 days. The AP_8_ was 0.07 at 30 days and reached 0.11 at 360 days, a 5.6-fold change over the autism rate.

### Performance in Subgroups

**Figure 4.**
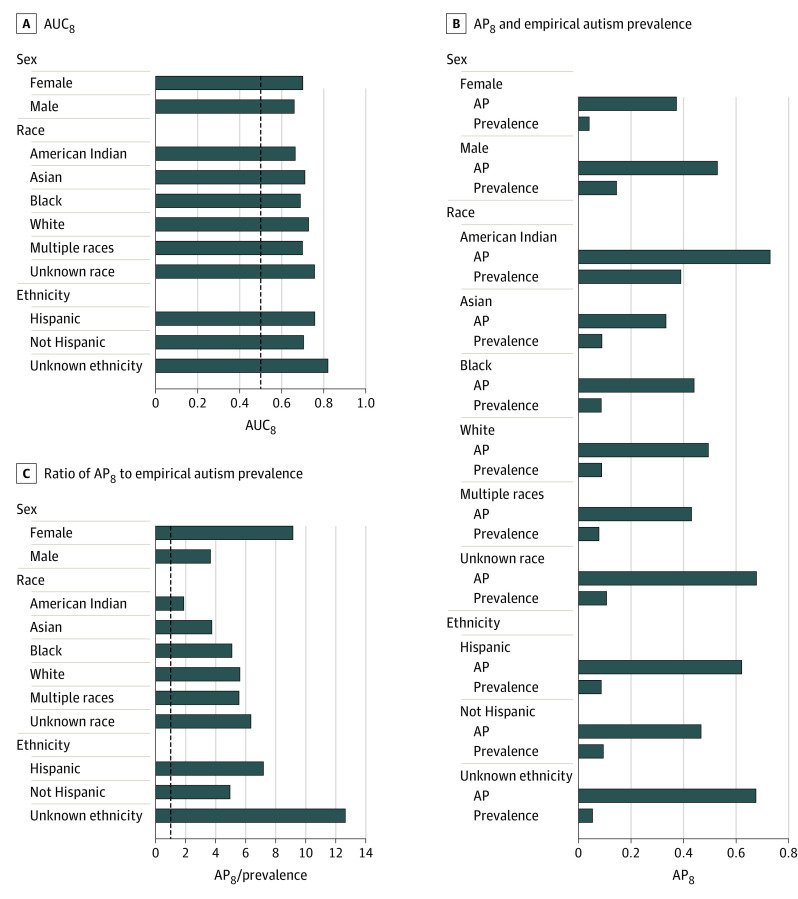
Model Prediction Performance by Sex, Race, and Ethnicity Performance when discriminating between children later diagnosed with autism and children not diagnosed through age 8 years was stratified by sex, race, and ethnicity. From left to right, the area under the receiver operating characteristic curve for individuals followed up through age 8 years (AUC_8_), the average (mean) positive predictive value for individuals followed up through age 8 years (AP_8_), and the ratio of AP_8_ to autism prevalence (AP_8_/prevalence) in each group are shown. Dashed vertical lines indicate performance associated with random guessing (ie, no information).

Differences in model prediction performance were assessed by sex, race, and ethnicity (Figure 4). The sensitivity of the observed differences in performance between racial groups to the required follow-up threshold *t* is summarized in eFigure 10 in Supplement 1. Differences in model prediction performance were also assessed by low birth weight (eFigure 11 in Supplement 1), and EHR system in use at the time of data collection (eFigure 12 and eTable 4 in Supplement 1). Complete results are provided in the eResults in Supplement 1.

### Feature Importance

**Figure 5.**
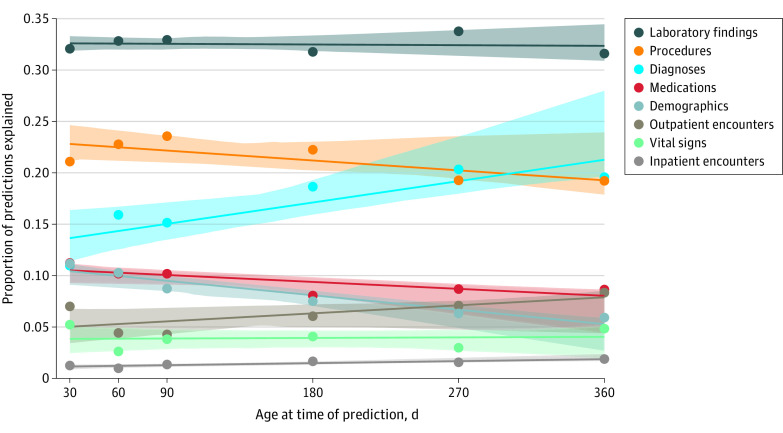
Feature Importance by Predictor Group The mean influence of each predictor (diagnosis codes, procedure codes, laboratory measurements, medications, vital signs, and encounter details) on model predictions (ie, feature importance) at 30, 60, 90, 180, 270, and 360 days was quantified by calculating the mean absolute value of all Shapley additive explanation values for that predictor on individuals in the test set. Values were then summed across all predictors in each predictor group (eg, diagnoses) to quantify the total effect of that group on predictions over time. Shaded areas represent 95% CIs.

The importance of different feature groups over time is presented in Figure 5. Complete results (eResults), including specific individual features at each time point (eFigures 13-15), effect of training phenotype (eFigure 16), and model-predicted risk in the secondary evaluation set (eFigure 17), are presented in Supplement 1.

## Discussion

Identification of autistic children early in childhood is necessary to ensure access to appropriate services and early behavioral support. Previously, we observed that autism diagnosis is associated with distinctive patterns of health care use before age 1 year, leading us to hypothesize that information documented in the EHR during routine care would be sufficient to detect autism by age 1 year or earlier. The results of this diagnostic study conducted in a large academic medical center suggest that EHR-based autism prediction reaches a clinically meaningful level of accuracy as early as 30 days after birth. We observed that almost half (45.5%) of autistic children can be identified at 30 days while maintaining high specificity (90.0%). The AP increased as data accumulated over the first year; therefore, even at very high-specificity operating points (97.0% at each time point; 94.3% overall), 38.8% of autistic children could be identified before age 1 year. However, increases in performance over time were smaller than hypothesized, which may suggest that relevant information was captured less consistently beyond 30 days or that it was present only in clinical notes rather than the structured fields available to our model. In contrast with existing screening tools, such as the M-CHAT, EHR-based autism detection takes place at a much earlier age (≥30 days) and is entirely passive, meaning that it does not require any data collection other than that which already takes place during routine care.

The results of this study were obtained not only at a very early age but also in a health system population with high rates of medical complexity and other neurodevelopmental conditions. Motivated by findings that co-occurring ADHD is associated with delays in autism diagnosis,^27,32^ a major emphasis of this work was to assess whether autism detection remained effective in subgroups of autistic children later diagnosed with ADHD or other neurodevelopmental conditions. Although the model’s ability to detect autism was lower in these groups compared with others, performance remained strong overall, particularly at high-specificity operating points likely to be required in clinical practice to maintain acceptable PPV. At such operating points (90.0%-97.0% specificity), contrary to our hypotheses, the sensitivity of autism detection was highest among children with other neurodevelopmental conditions and was similar between children with and without ADHD.

Additional subgroup analyses showed that good performance was not limited to particular demographic groups. The results of this study suggest that model-based autism detection (AUC_8_) was more effective in girls than in boys. It was also effective across all races and ethnicities, but performance was higher in White children than in Black children despite similar numbers and rates of autism diagnoses between groups (ie, 323 of 14 549 Black children [2.2%] vs 369 of 18 871 White children [2.0%]). The effect of premature birth on model predictions is difficult to quantify directly due to the large number of diagnosis codes, procedure codes, and other features associated with prematurity that were available to the model. However, performance changed very little when excluding individuals whose earliest recorded weight was below the fifth percentile, suggesting that the model was not simply equating autism with premature birth. Differences in model-based autism detection (AUC_4_) before vs after DUHS adoption of the Epic EHR platform may be due to the greater detail and fidelity of EHR variables after this transition.

The individual feature importance statistics presented should be interpreted with caution. These results are associative only and do not suggest that a causal relationship is present. Furthermore, our models incorporate hundreds of predictors, many of which are correlated. This makes it likely that training with a different random sample would yield substantially different feature importance results. To capture the importance of gastrointestinal-related conditions, for example, one model might place greater weight on gastrointestinal diagnoses, while another might place greater weight on gastrointestinal-related procedures. In contrast, we believe that the broad trends highlighted in Figure 5 are more likely to generalize to other health systems and populations. As time goes on, the model relies less on demographics and perinatal procedures, and it instead relies more on diagnosis codes as co-occurring conditions manifest and are recognized.

An important secondary aim of this study has been to understand how performance is affected by the specific computable phenotype used to label autism diagnoses. Obtaining accurate labels can be time and labor intensive and, while critical for accurate model evaluation, having accurate labels during training is important only insofar as it improves performance. Across all time points (30-360 days), our results showed that using a weak version of our computable phenotype during training—namely, defining all children with 1 or more documented autism codes as autism case patients—was just as effective as training based on a stronger, previously validated phenotype.^8^ Determining whether this finding extends to other conditions or settings may be an important direction for future work.

Our medical record review subpopulation was important not only to validate our computable phenotype within the DUHS but also to evaluate model-based autism detection among individuals with positive M-CHAT-R/F status. Interestingly, model-predicted risk scores of autistic children identified by medical record review but not meeting our computable phenotype were similar to those of control participants at 30 days. By 360 days, on the other hand, their scores were more similar to the scores of the remaining autistic children who also met our computable phenotype. Although our primary evaluation shows that identification of individuals meeting the computable phenotype was similar at 30 and 360 days, this finding suggests that identification of true cases increases over time. Overall, results in this subpopulation suggest that model-based autism detection can help determine which children with positive M-CHAT-R/F status will be diagnosed.

We are working to further refine and deploy our models within the DUHS, integrate them within clinical workflows, and test the effect of presenting model predictions to parents and providers. To understand potential benefits of these models, it will be important to compare predictions with scores from other screening tools, including the M-CHAT, to assess whether they identify similar vs distinct subpopulations of autistic children. Future work will also explore whether clinical notes can be leveraged to further boost performance.

### Limitations

All analyses in this diagnostic study were based on DUHS data. These results may not generalize to settings with different demographic or population health characteristics or to different EHR systems or data models. Our models can be applied only if EHR data are available in the first year of life, and it is unlikely that results could be adapted to settings where EHRs are not routinely available, including among individuals without access to health care. Our analyses of model performance were based on a validated computable phenotype rather than certain autism diagnoses. This approach is vulnerable to known biases in diagnosis, including underdiagnosis of girls and women. Further, diagnoses occurring outside of the DUHS may have been missed. Information about race and ethnicity was collected from 2006 to 2021 across multiple EHR systems and may not have been collected according to current best practices (ie, patient- or parent-report depending on age). Finally, intellectual disability was underrepresented likely because diagnoses are often made at a later age, after neuropsychological or psychoeducational testing can be completed.

## Conclusions

In this diagnostic study of an autism screening test, EHR-based early autism detection was effective by age 30 days and provided information about autism likelihood that was complementary to the M-CHAT. The results suggest that EHR-based monitoring should be integrated with the M-CHAT, other caregiver surveys, and other screening tools to improve the accuracy of early autism screening.

## Notes

Supplement 1.
eMethods.

eResults.

eReferences
**eTable 1.** Demographics and Rates of Other Neurodevelopmental Conditions **eTable 2.** Performance Measures Over Time **eTable 3.** Diagnosis Codes for Computable Phenotypes **eTable 4.** Missingness Rate by Predictor Group **eFigure 1.** Selection of Autism Case Patients and Control Participants **eFigure 2.** Number of Encounters Over Time **eFigure 3.** Diagnosis Timing **eFigure 4.** Prediction Performance by Age Among Children Without Other Neurodevelopmental Conditions **eFigure 5.** Sensitivity to Follow-up Threshold for 30-Day Models **eFigure 6.** Sensitivity to Follow-up Threshold for 360-Day Models **eFigure 7.** Operating Points **eFigure 8.** Calibration Curves **eFigure 9.** Prediction Performance at 360 Days for Individuals With and Without Other Neurodevelopmental Conditions **eFigure 10.** Sensitivity of Prediction Performance Stratified by Race to Follow-up Threshold **eFigure 11.** Prediction Performance Stratified by Low Birth Weight **eFigure 12.** Prediction Performance by Electronic Health Record System (Legacy vs Epic) **eFigure 13.** Individual Feature Importance for the 30- and 360-Day Models **eFigure 14.** Individual Feature Importance for the 60- and 90-Day Models **eFigure 15.** Individual Feature Importance for the 180- and 270-Day Models **eFigure 16.** Effect of Training Phenotype **eFigure 17.** Model-Predicted Risk in the Secondary Evaluation Set

Supplement 2.
Data Sharing Statement
