# Supplementary material for: Predictive Value of Early Autism Detection Models Based on Electronic Health Record Data Collected Before Age 1 Year
Source: JAMA Netw Open. 2023 Feb 2;6(2):e2254303. doi: 10.1001/jamanetworkopen.2022.54303 (PMC9896305; doi:10.1001/jamanetworkopen.2022.54303)
Supplement: Supplement 1. — eMethods. eResults. eReferences eTable 1. Demographics and Rates of Other Neurodevelopmental Conditions eTable 2. Performance Measures Over Time eTable 3. Diagnosis Codes for Computable Phenotypes eTable 4. Missingness Rate by Predictor Group eFigure 1. Selection of Autism Case Patients and Control Participants eFigure 2. Number of Encounters Over Time eFigure 3. Diagnosis Timing eFigure 4. Prediction Performance by Age Among Children Without Other Neurodevelopmental Conditions eFigure 5. Sensitivity to Follow-up Threshold for 30-Day Models eFigure 6. Sensitivity to Follow-up Threshold for 360-Day Models eFigure 7. Operating Points eFigure 8. Calibration Curves eFigure 9. Prediction Performance at 360 Days for Individuals With and Without Other Neurodevelopmental Conditions eFigure 10. Sensitivity of Prediction Performance Stratified by Race to Follow-up Threshold eFigure 11. Prediction Performance Stratified by Low Birth Weight eFigure 12. Prediction Performance by Electronic Health Record System (Legacy vs Epic) eFigure 13. Individual Feature Importance for the 30- and 360-Day Models eFigure 14. Individual Feature Importance for the 60- and 90-Day Models eFigure 15. Individual Feature Importance for the 180- and 270-Day Models eFigure 16. Effect of Training Phenotype eFigure 17. Model-Predicted Risk in the Secondary Evaluation Set [file jamanetwopen-e2254303-s001.pdf]

Supplementary Online Content

Engelhard MM, Henao R, Berchuck SI, et al. Predictive value of early autism detection models based on electronic health record data collected before age 1 year. *JAMA Netw Open*. 2023;6(2):e2254303. doi:10.1001/jamanetworkopen.2022.54303

eMethods

eResults

eReferences

eTable 1. Demographics and Rates of Other Neurodevelopmental Conditions

eTable 2. Performance Measures Over Time

eTable 3. Diagnosis Codes for Computable Phenotypes

eTable 4. Missingness Rate by Predictor Group

eFigure 1. Selection of Autism Case Patients and Control Participants

eFigure 2. Number of Encounters Over Time

eFigure 3. Diagnosis Timing

eFigure 4. Prediction Performance by Age Among Children Without Other Neurodevelopmental Conditions

eFigure 5. Sensitivity to Follow-up Threshold for 30-Day Models

eFigure 6. Sensitivity to Follow-up Threshold for 360-Day Models

eFigure 7. Operating Points

eFigure 8. Calibration Curves

eFigure 9. Prediction Performance at 360 Days for Individuals With and Without Other Neurodevelopmental Conditions

eFigure 10. Sensitivity of Prediction Performance Stratified by Race to Follow-up Threshold

eFigure 11. Prediction Performance Stratified by Low Birth Weight

eFigure 12. Prediction Performance by Electronic Health Record System (Legacy vs Epic)

eFigure 13. Individual Feature Importance for the 30- and 360-Day Models

eFigure 14. Individual Feature Importance for the 60- and 90-Day Models

eFigure 15. Individual Feature Importance for the 180- and 270-Day Models

eFigure 16. Effect of Training Phenotype

eFigure 17. Model-Predicted Risk in the Secondary Evaluation Set

This supplementary material has been provided by the authors to give readers additional information about their work.

eMethods

Secondary evaluation by chart review

EHR data from a sub-sample of 309 study participants with positive MCHAT-R/F status or an eventual diagnosis of autism were manually examined and coded by members of the study team. These individuals' EHR data were retrospectively extracted and coded in 6-month time increments starting from 18-months of age through the latest visit available. Records were also combined with those from an ongoing prospective study at Duke that includes patients from our study population to ensure correct diagnostic codes from cross-referencing study evaluations as well as EHR data. Coded data included caregiver and provider concerns, documentation of the date/timing of referral to autism evaluation, evaluation status, and diagnosis at any institution.

Diagnosis status in this sub-sample was compared with our computable phenotype to evaluate correspondence between the two, including the sensitivity and specificity of the computable phenotype. Additionally, model performance was evaluated for individuals in this sub-sample who were also in our test set.

EHR variables and preprocessing

Prediction models were based on data collected by age 30, 60, 90, 180, 270, and 360 days, respectively. For example, the input features to the 30-day models were derived from patient-associated EHR variables collected before the age of 30 days. Features were derived from demographic information (sex, race, ethnicity) as well as documented diagnosis codes, procedure codes, laboratory measurements, medications, vital signs, and encounter details. Diagnosis and procedure codes were grouped using Clinical Classification Software (CCS) Level 1 categories.[1] Medications were grouped using the National Drug Code (NDC) Directory. Laboratory measurements were grouped manually. Encounters were first identified as inpatient or outpatient, then grouped by the clinical service or specialty associated with that encounter. For each of the aforementioned grouped categorical variables, features for each patient were derived by counting the number of occurrences of each group (*e.g.*, the number of encounters with each possible clinical service or specialty) within the data collection window (*e.g.*, before age 30 days), and log-transforming the result. All vital signs were aggregated across the data collection window by taking the maximum, minimum, and mean values. If a particular measurement was not collected within the data collection window, its maximum, minimum, and mean values were imputed to median values across the training set. The total number of predictors ranged from 609 at 30 days to 898 at 360 days.

Model development and evaluation

Data were divided at random into a training set (60%) used to develop models and a test set (40%) used to evaluate the performance of the final model.

During model development, 5-fold cross-validation was used to tune hyperparameters and select between models. Three types of models were explored during model development: (a) L2-regularized Cox proportional hazards (Cox-PH) models;[2] (b) gradient boosting survival analysis models trained to optimize the Cox-PH partial likelihood,[3] and (c) random survival forest models.[4] Hyperparameters tuned during model development included the strength of the L2 penalty (Cox-PH), the number of features per estimator and dropout rate (gradient boosting models), and the number of estimators and features per split (random survival forest models). All models were defined and trained using scikit-survival[5] in Python (v3.7). Among all models, the model with highest average concordance index (see Performance Measures) during cross-validation was selected as the final model, then applied to the test set and evaluated.

Additional models were developed in parallel to explore the sensitivity of the model development process and resulting performance to (a) variations in the population used to train the models, and (b) the computable phenotype used to identify autism cases. To explore the former, we altered the controls in the training and validation sets to exclude individuals later diagnosed with neurodevelopmental conditions other than autism,

including ADHD and intellectual disability. To explore the latter, we altered the cases in the training and validation sets to include all individuals with at least one documented autism code. We refer to this as the weak phenotype in contrast to the stronger phenotype previously described (see Case Definitions and Cohort Selection).

Performance measures

The following measures were used during model development or model evaluation. Please note that in this section and elsewhere, we use *risk* in a mathematical sense to denote a patient's instantaneous probability of diagnosis per unit time given that they were not diagnosed previously. The term is not meant to imply that autism or an autism diagnosis are negative.

Area under the receiver operating characteristic curve (AUC) and AUC_t

The area under the receiver operating characteristic curve (AUC) assesses the model's ability to distinguish between cases and controls – in this case, children later diagnosed with autism and children not diagnosed. It quantifies the probability that model-predicted risk for a randomly selected child who was later diagnosed with autism is higher than model-predicted risk for a randomly selected child who was not, which is equal to the area under the sensitivity versus specificity curve. Since lifetime diagnosis status is highly uncertain for patients with short follow-up, we primarily report the AUC_t rather than the AUC, where AUC_t is defined as the AUC when limiting negative cases to individuals followed for at least *t* years.

Average positive predictive value (AP) and AP_t

The average positive predictive value (PPV), also known as the average precision (AP), is a conservative estimate of area under the PPV versus sensitivity curve. It quantifies the average PPV across a range of prediction thresholds with varying sensitivity. Compared to the AUC, the AP is preferred when the number of cases is much smaller than the number of controls. Similar to the AUC, we primarily report the AP_t rather than the AP, where AP_t is defined as the AP when limiting negative cases to individuals followed for at least *t* years.

Concordance Index (CI)

The concordance index (CI) quantifies the probability that model-predicted risk for a randomly selected pair of individuals is consistent with observed diagnosis and censoring times.[6] A given pair of individuals contributes to the CI only if (a) diagnosis is observed for both of them, or (b) diagnosis is observed at age *t* in only one individual, but the other is followed beyond age *t* without being diagnosed.

Although the concordance index was used to select our final prediction model, as previously described, the AUC_t and AP_t were chosen as our primary evaluation measures due to their higher clinical relevance and interpretability. To contextualize these measures, we also report the corresponding effective prevalence after controls followed for fewer than *t* years were excluded. The sensitivity of these measures to the threshold *t* was explored by calculating AUC_t and the corresponding receiver operating characteristic (i.e., sensitivity versus 1 - specificity) curve; and AP_t and the corresponding PPV versus sensitivity curve. The AUC_8 and AP_8 were explored in greatest depth; the selection of this cutoff reflects a compromise between (a) ensuring children have been followed long enough for diagnosis to be likely, were it to occur; and (b) maintaining a large evaluation sample covering a wide range of birth years. High sensitivity and high specificity operating points were selected to achieve 90% sensitivity and specificity, respectively, based on an 8-year follow-up threshold. 1-calibration[7], which is based on a Hosmer-Lemeshow test statistic, was used to quantify correspondence between model-predicted risk and true diagnosis rates for all models at ages 4 and 8 years.

All performance measures were evaluated on the full test set as well as in subgroups defined by demographic variables (sex, race, ethnicity) and two other factors of interest. The first of these factors, low birth weight, was designed to investigate the degree to which model predictions and performance were driven by factors related to premature birth. This factor was defined as positive for all individuals whose earliest recorded birth weight was below the 5th percentile based on World Health Organization growth charts available from the National Center for Health Statistics. The second of these factors indicates whether individuals were born before 2013. This factor was designed to investigate whether performance differed between individuals whose data was extracted from the current (2013 – present) DUHS EHR versus older, legacy systems.

© 2023 Engelhard MM et al. *JAMA Network Open*

eResults

Description of cohort

Cohort demographics and rates of each neurodevelopmental condition are compared between autism cases and controls in sTable 1. Among the autism cases, there were 738 males and 186 females (79.9% male). All four groups of neurodevelopmental conditions other than autism included in the analysis occurred at higher rates among the autism cases compared to the controls ($p < 0.001$): there were 266, 44, 7, and 766 autism cases with co-occurring ADHD, intellectual disability, genetic neurodevelopmental conditions, and other neurodevelopmental conditions, respectively.

There were group differences in the number of encounters between autism cases and controls throughout the first year of life (see sFigure 2). The median number of encounters before 30 days was 3 in both groups, but the 75th percentile was higher among autism cases (5) versus controls (4), and the difference between distributions was statistically significant (p=0.030). The number of encounters in all other windows (30-60 days, 60-90 days, 90-180 days, 180-270 days, 270-360 days) was higher among autism cases than controls (p<0.001).

Autism cases made up 2.0% of the individuals included in our analysis. Adjusting for right censoring, the estimated cumulative rate of autism diagnosis was 0.1% at age 2, 0.6% at age 3, 1.2% at age 4, 2.0% at age 6, 2.5% at age 8, 3.1% at age 10, and 3.4% at age 12 (see sFigure 3).

Prediction performance over time

Sensitivity to the length of required follow-up among controls is shown for the 30-day models (sFigure 5) and the 360-day models (sFigure 6). At 30 days, the AUC_t ranged from an AUC_4 of 0.688 to an AUC_{10} of 0.801, and the AP_t ranged from an AP_4 of 0.110 (2.3-fold increase over effective prevalence) to an AP_{10} of 0.530 (3.2-fold increase over effective prevalence). At 360 days, the AUC_t ranged from an AUC_4 of 0.701 to an AUC_{10} of 0.826, and the AP_t ranged from an AP_4 of 0.160 (3.4-fold increase over effective prevalence) to an AP_{10} of 0.606 (3.6-fold increase over effective prevalence). When varying the required follow-up length, the number of cases included in the evaluation (N=363) was unchanged, but the number of controls 7638, 6537, 5373, 4428, 3615, 2868, and 2173 at *t* values of 4 to 10 years, respectively.

Sensitivity and PPV at our high (90%)-specificity and very high (97%)-specificity operating points, as well as specificity and PPV at our high (90%)-sensitivity operating points, are summarized in sTable 2, while the operating points themselves are depicted in sFigure 7. At the high-specificity operating points, sensitivity ranged from 0.452 at 60 days to 0.482 at 270 days, and PPV ranged from 0.226 at 90 days to 0.239 at 270 days. At the very high-specificity operating points, sensitivity ranged from 0.256 at 90 days to 0.292 at 30 days, and PPV ranged from 0.360 at 90 days to 0.393 at 30 days. Finally, at the high-sensitivity operating points, specificity ranged from 0.362 at 30 days to 0.396 at 270 days, and PPV ranged from to 0.085 at 30 days to 0.089 at 270 days.

Model calibration at age 4 and 8 years is shown for the 30-day and 360-day models in sFigure 8. The corresponding 1-calibration statistics[7] are included in sTable 2.

Prediction among those with other neurodevelopmental conditions

Figure 3 further illustrates the effect of other neurodevelopmental conditions on correct identification of autism cases and controls over time. At high specificity operating points, detection of autism cases was highest among those with another neurodevelopmental condition other than ADHD both at 30 days (56.1%) and by 360 days (68.2%). Approximately half (46.7% and 50.0%, respectively) of those with comorbid ADHD and those without any other neurodevelopmental condition were detected by 360 days. False positive rates at 30 days were highest among controls with a neurodevelopmental condition other than ADHD (16.7%) followed by those with ADHD (13.6%) and those with neither (7.2%). Across the full test set, 59.8% of the autism cases

© 2023 Engelhard MM et al. *JAMA Network Open*

were detected by 360 days (*i.e.*, sensitivity of combined models), and 81.5% of controls were predicted negative at all time points (*i.e.*, specificity of combined models).

At *very* high specificity operating points, detection of autism cases was again highest among those with another neurodevelopmental condition other than ADHD both at 30 days (38.8%) and by 360 days (51.9%). Rates of detection at 30 days were higher for those with co-occurring ADHD (16.2%) compared to those without any other neurodevelopmental condition (13.6%), but this trend reversed by 360 days (19.0% versus 22.8%, respectively). False positive rates at 30 days were highest among controls with a neurodevelopmental condition other than ADHD (6.7%) followed by those with ADHD (4.1%) and those with neither (1.5%). Across the full test set, 38.8% of the autism cases were detected by 360 days (*i.e.*, sensitivity of combined models), and 94.3% of controls were predicted negative at all time points (*i.e.*, specificity of combined models).

Performance in subgroups

AUC_8 was higher in females (0.794) than in males (0.748) (see Figure 4). AP_8 was higher in males (0.527) than in females (0.372), fold increase in AP_8 over autism prevalence was higher in females (9.1) than in males (3.7). Among all racial groups represented in the test set (>15 individuals), AUC_8 ranged from 0.753 (American Indian / Alaskan Native) to 0.857 (Unknown Race), and was higher among White individuals (0.825) than among Black (0.781) or Asian (0.805) individuals (see sFigure 10). AP_8 and the fold increase in AP_8 over autism prevalence were lowest among Asian individuals (0.332 and 3.7, respectively). Both AUC_8 and AP_8 were higher in Hispanic individuals (0.859 and 0.620, respectively) than in non-Hispanic individuals (0.798 and 0.466, respectively; see sFigure 11).

There were 29 autism cases (8.0%) and 1030 controls (5.8%) in the test set (X^2 p-value=0.105) whose earliest recorded weight was below the 2nd percentile. AUC_8 was higher among individuals whose earliest recorded weight was below the 2nd percentile (0.913) compared to others (0.801), but the fold increase in AP_8 over autism prevalence was lower (3.5 versus 5.3). These trends were similar among the 38 autism cases (10.5%) and 1640 controls (9.3%) in the test set (X^2 p-value=0.497) whose earliest recorded weight was below the 5th percentile (see sFigure 12). AUC_8 was higher among individuals whose earliest recorded weight was below the 5th percentile (0.883) compared to others (0.798), but the fold increase in AP_8 over autism prevalence was similar (4.7 versus 4.8).

Performance was higher among individuals born after DUHS adoption of the Epic EHR platform (AUC_4=0.778, AP_4=0.091, AP_4 / effective prevalence=2.3) compared to those born earlier (AUC_4=0.677, AP_4=0.265, AP_4 / effective prevalence=4.6; see sFigure 13).

Feature importance

Among the different feature (*i.e.*, predictor) groups, laboratory measurements had the greatest total influence on model predictions across all time points, ranging from 31.6% of predictions explained at 180 days to 33.8% at 270 days (see Figure 5). Procedures had the second greatest influence, peaking at 23.6% at 90 days and declining to 19.2% at 360 days. Diagnoses became more important over time (*r*=0.857; *p*=0.029), accounting for 11.0% of predictions at 30 days up to 19.6% at 360 days. Demographics became less important over time (*r*=-0.952, *p*=0.003), accounting for 11.2% of predictions at 30 days down to 5.9% at 360 days. Inpatient encounters also became more important over time (*r*=0.881, *p*=0.020). Other changes over time were not statistically significant (*p*>0.05).

The importance of specific predictors at each time point (see sFigures 14-16) show that on average, the single most influential predictor was the count of blood glucose measurements ($\mu_{|\mathrm{SHAP}|}$=0.038) followed by the count of basic metabolic panels ($\mu_{|\mathrm{SHAP}|}$=0.034), male sex ($\mu_{|\mathrm{SHAP}|}$=0.030), the count of diagnosis codes associated with administrative and social admissions (CCS category 255; $\mu_{|\mathrm{SHAP}|}$=0.020), the count of complete blood counts ($\mu_{|\mathrm{SHAP}|}$=0.017), and the count of other diagnostic procedures (*e.g.*, interview, evaluation, consultation; $\mu_{|\mathrm{SHAP}|}$=0.015).

© 2023 Engelhard MM et al. *JAMA Network Open*

Following administrative and social admissions, the next 5 diagnosis categories with highest feature importance were CCS categories 256 (medical examination / evaluation; $\mu_{|\mathrm{SHAP}|}$=0.015), 218 (liveborn; $\mu_{|\mathrm{SHAP}|}$=0.006), 91 (other eye disorders; $\mu_{|\mathrm{SHAP}|}$=0.005), 224 (other perinatal conditions; $\mu_{|\mathrm{SHAP}|}$=0.004), and 219 (short gestation; $\mu_{|\mathrm{SHAP}|}$=0.003).

Following other diagnostic procedures, the next 5 procedure categories with highest feature importance were CCS categories 235 (other laboratory; $\mu_{|\mathrm{SHAP}|}$=0.011), 183 (routine chest x-ray; $\mu_{|\mathrm{SHAP}|}$=0.010), 231 (other therapeutic procedures; $\mu_{|\mathrm{SHAP}|}$=0.008), 216 (respiratory intubation and mechanical ventilation; $\mu_{|\mathrm{SHAP}|}$=0.007), and 218 (psychological and psychiatric evaluation; $\mu_{|\mathrm{SHAP}|}$=0.007).

Effect of diagnosis criteria

Performance was also evaluated for a second set of models trained with the weak autism phenotype (≥1 autism-related ICD code; see Model Development and Evaluation). In addition to the 363 individuals in the test set meeting autism diagnosis criteria, there were 56 individuals in the test set that did not meet criteria but did satisfy the weak phenotype. Risk predicted by models trained on the weak phenotype was strongly correlated with risk predicted by our primary models across all time points (ρ=0.959, 0.943, 0.936, 0.944, 0.927, and 0.937 at 30, 60, 90, 180, 270, and 360 days, respectively).

The two sets of models had similar performance (see sFigure 17) when discriminating between autism cases and controls (p>0.5) except at 30 days, when discrimination performance was higher for the model trained with the weak phenotype (AUC_8=0.813) compared to the corresponding, primary 30-day model (AUC_8=0.794; p=0.001). The two sets of models also had similar performance when discriminating between non-autism cases meeting the weak phenotype and controls (p>0.5) except at 30 days, when discrimination performance was higher for the model trained with the weak phenotype (AUC_8=0.833) compared to the corresponding, primary 30-day model (AUC_8=0.791; p=0.025). None of the models trained with either phenotype was able to effectively distinguish between autism cases and those not meeting full criteria but satisfying the weak phenotype (AUC_8<0.512 for all models), and differences between models trained with the weak versus full phenotype were not statistically significant (p>0.05).

Prediction of cases identified by chart review

Of the 309 participants evaluated by chart review, 74 were later determined to have an autism diagnosis. Of these 74, 52 met our autism computable phenotype (sensitivity=70.3%), and an additional 6 met the weak phenotype (≥1 autism-related diagnosis code). Of the remaining 236 without an autism diagnosis, only 3 met our computable phenotype (specificity=98.7%).

A total of 79 of these 309 participants were in the test set, including 23 determined by chart review to have an autism diagnosis. Model AUC when discriminating between these 23 cases and the other 56 controls was 0.630 at 30 days, 0.643 at 60 days, 0.666 at 90 days, 0.609 at 180 days, 0.585 at 270 days, and 0.605 at 360 days.

At 30 days (see sFigure 18), model-predicted risk among individuals determined to have an autism diagnosis was higher for those who also satisfied the computable phenotype compared to those who did not (p=0.036). However, at 360 days, this was no longer true: among those with an autism diagnosis, model-predicted risk was higher for those not satisfying the computable phenotype (p=0.039).

© 2023 Engelhard MM et al. *JAMA Network Open*

eReferences

1. Elixhauser A, Steiner C, Palmer L. *Clinical Classification Software (CCS), 2014. US Agency for Healthcare Research and Quality. 2014*.; 2014.

2. Cox DR. Regression models and life-tables. *Journal of the Royal Statistical Society: Series B (Methodological)*. 1972;34(2):187-202.

3. Hothorn T, Bühlmann P, Dudoit S, Molinaro A, Van Der Laan MJ. Survival ensembles. *Biostatistics*. 2006;7(3):355-373.

4. Ishwaran H, Kogalur UB, Blackstone EH, Lauer MS. Random survival forests. *The annals of applied statistics*. 2008;2(3):841-860.

5. Pölsterl S. scikit-survival: A Library for Time-to-Event Analysis Built on Top of scikit-learn. *J Mach Learn Res*. 2020;21(212):1-6.

6. Harrell Jr FE, Lee KL, Califf RM, Pryor DB, Rosati RA. Regression modelling strategies for improved prognostic prediction. *Statistics in medicine*. 1984;3(2):143-152.

7. Haider H, Hoehn B, Davis S, Greiner R. Effective Ways to Build and Evaluate Individual Survival Distributions. *J Mach Learn Res*. 2020;21(85):1-63.

© 2023 Engelhard MM et al. *JAMA Network Open*

eTable 1. Demographics and Rates of Other Neurodevelopmental Conditions

Variable	Value	Autism	Control	p-value
Total	*N*	924	44,156	
Sex	*Female*	186 (20.1%)	21302 (48.2%)	**<0.001**
	Male	738 (79.9%)	22852 (51.8%)	
	Unknown Sex	0 (0.0%)	2 (0.0%)	
Race	*American Indian or Alaskan Native*	8 (0.9%)	243 (0.6%)	0.293
	Asian	32 (3.5%)	1846 (4.2%)	0.319
	Black	323 (35.0%)	14226 (32.2%)	0.084
	Multiracial	40 (4.3%)	2248 (5.1%)	0.333
	Native Hawaiian or Pacific Islander	3 (0.3%)	96 (0.2%)	0.738
	Unknown Race	149 (16.1%)	6995 (15.8%)	0.851
	White	369 (39.9%)	18502 (41.9%)	0.244
Ethnicity	*Hispanic*	137 (14.8%)	6606 (15.0%)	0.947
	Not Hispanic	744 (80.5%)	35187 (79.7%)	0.561
	Unknown Ethnicity	43 (4.7%)	2363 (5.4%)	0.390
ADHD	*Present*	266 (28.8%)	1953 (4.4%)	**<0.001**
	Absent	658 (71.2%)	42203 (95.6%)	
Intellectual Disability	*Present*	44 (4.8%)	122 (0.3%)	**<0.001**
	Absent	880 (95.2%)	44034 (99.7%)	
Other Neurodevelopmental Condition	*Present*	766 (82.9%)	9562 (21.7%)	**<0.001**
	Absent	158 (17.1%)	34594 (78.3%)	
Genetic Condition	*Present*	7 (0.8%)	19 (0.0%)	**<0.001**
	Absent	917 (99.2%)	44137 (100.0%)	

© 2023 Engelhard MM et al. *JAMA Network Open*

eTable 2. Performance Measures Over Time

	Data Collection Threshold					
Performance Measure	30 days	60 days	90 days	180 days	270 days	360 days
Concordance Index	0.765	0.773	0.766	0.773	0.778	0.774
AUC_8	0.764	0.770	0.762	0.769	0.774	0.770
AP_8	0.235	0.255	0.239	0.249	0.266	0.259
Sensitivity at 90% specificity	0.455	0.452	0.455	0.457	0.482	0.477
PPV at 90% specificity	0.230	0.227	0.226	0.230	0.239	0.232
Sensitivity at 97% specificity	0.292	0.287	0.256	0.273	0.287	0.270
PPV at 97% specificity	0.393	0.384	0.360	0.376	0.382	0.370
Specificity at 90% sensitivity	0.362	0.380	0.385	0.390	0.396	0.382
PPV at 90% sensitivity	0.085	0.087	0.088	0.089	0.089	0.088
One-calibration p-value at 4 years	0.094	0.019	0.329	0.767	0.027	0.150
One-calibration p-value at 8 years	<0.001	0.002	<0.001	<0.001	<0.001	0.019

© 2023 Engelhard MM et al. *JAMA Network Open*

eTable 3. Diagnosis Codes for Computable Phenotypes

Condition	ICD-9	ICD-10
Autism	299.00, 299.01, 299.80, 299.81, 299.90	F84.0, F84.8, F84.5, F84.9
ADHD	314, 314.0, 314.01, 314.1, 314.2, 314.8, 314.9	F90.0, F90.1, F90.2, F90.8, F90.9
Intellectual Disability	317.0, 318.0, 318.1, 318.2, 319.0	F70.0, F71.0, F72.0, F73.0, F78.0, F79.0
Genetic Neurodevelopmental Condition	759.83	F84.2, G40.80, Q87.11, Q93.5, Q93.82, Q99.2
Other Neurodevelopmental Condition	307.00, 307.20, 307.21, 307.22, 307.23, 307.9, 313.89, 315.00, 315.01, 315.02, 315.09, 315.1, 315.2, 315.31, 315.32, 315.35, 315.39, 315.4, 315.5, 315.8, 780.99, 781.2, 781.3, 783.40, 783.42, 784.59	F80.0, F80.1, F80.2, F80.81, F80.82, F80.89, F80.9, F81.0, F81.2, F81.8, F81.89, F81.9, F82.0, F88.0, F94.8, F95.0, F95.1, F95.2, F95.8, F95.9, R26.9, R27.9, R29.898, R47.9, R48.2, R62, R62.0, R62.5, Z73.4

© 2023 Engelhard MM et al. *JAMA Network Open*

eTable 4. Missingness Rate by Predictor Group. Values show the proportion of children without any available data (e.g. no laboratory measurements) in a given predictor group. Results are shown for children born prior to 2013 versus in 2013 or later, after DUHS transitioned to the Epic EHR platform.

30-day Data Collection Threshold		
	Pre-Epic	Post-Epic
Diagnoses	7.7%	5.3%
Inpatient Encounters	17.4%	24.0%
Labs	11.6%	17.2%
Medications	93.6%	26.5%
Outpatient Encounters	25.0%	19.5%
Procedures	3.0%	4.5%
Vitals	50.9%	5.9%
360-day Data Collection Threshold		
Diagnoses	2.8%	4.2%
Inpatient Encounters	12.5%	17.2%
Labs	9.9%	14.2%
Medications	70.2%	12.2%
Outpatient Encounters	9.2%	6.5%
Procedures	2.8%	4.2%
Vitals	31.4%	0.5%

eFigure 1. Selection of Autism Case Patients and Control Participants

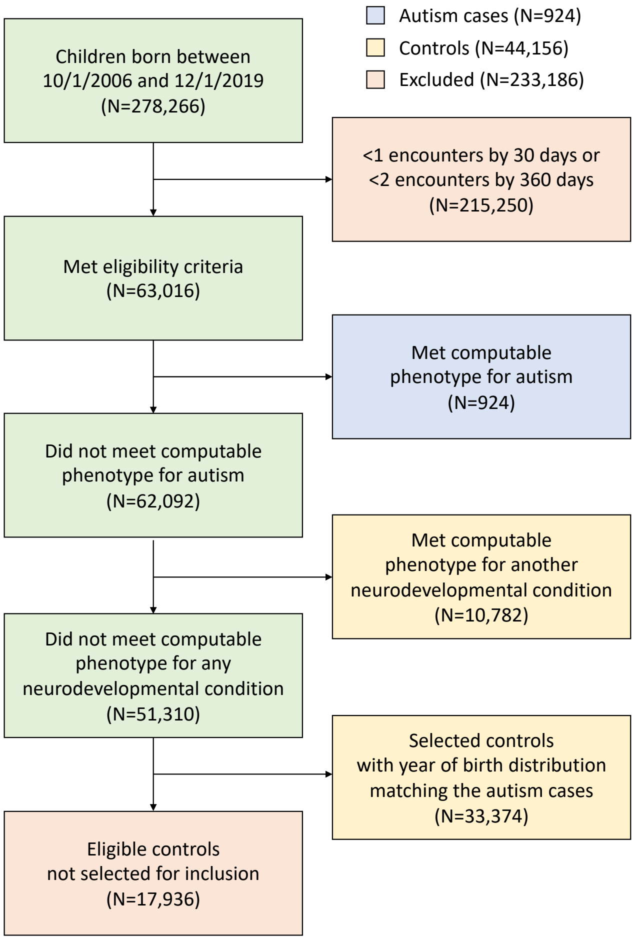

© 2023 Engelhard MM et al. *JAMA Network Open*

eFigure 2. Number of Encounters Over Time. The figure shows the number of encounters by 30, 60, 90, 180, 270, and 360 days among children later meeting our autism computable phenotype and all other children meeting inclusion criteria. The lines indicate the median number of encounters, and the shading indicates the 25th and 75th percentiles, respectively.

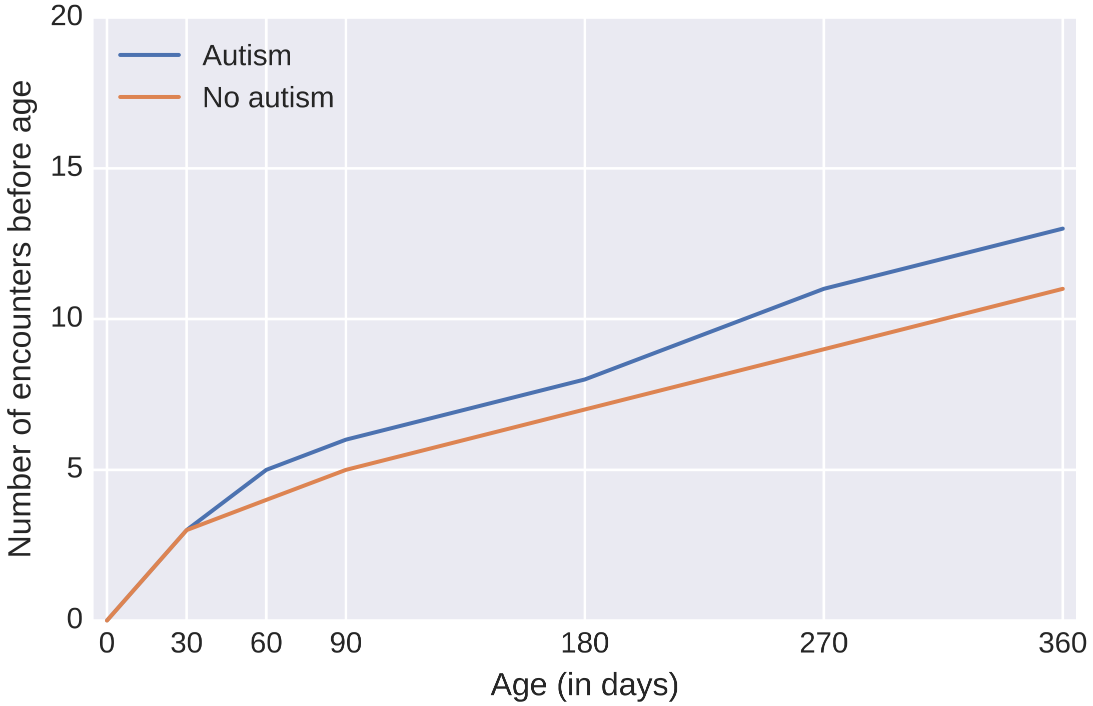

© 2023 Engelhard MM et al. *JAMA Network Open*

eFigure 3. Diagnosis Timing. Nelson-Aalen estimate of the cumulative autism diagnosis rate among individuals meeting inclusion criteria.

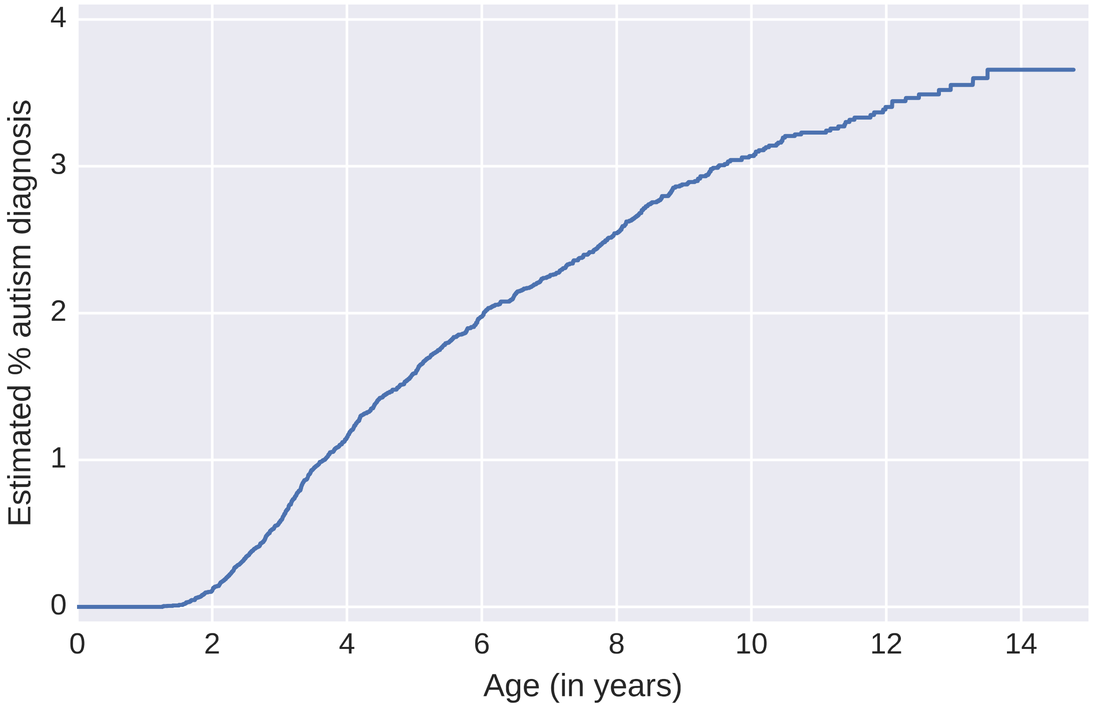

© 2023 Engelhard MM et al. *JAMA Network Open*

eFigure 4. Prediction Performance by Age Among Children Without Other Neurodevelopmental Conditions. The figures show autism prediction performance based on data collected from birth through 30 days, 60 days, 90 days, 180 days, 270 days, and 360 days, respectively. Cases were defined as children later meeting our autism computable phenotype, but in contrast to Figure 1, controls were defined as children followed through age 8 but not meeting our phenotype for autism or any other neurodevelopmental condition. The top panels summarize performance by age via the area under the receiver operating characteristic curve (top left) and average positive predictive value (top right). The bottom panels show the tradeoff between sensitivity and specificity (bottom left) and sensitivity and positive predictive value (bottom right).

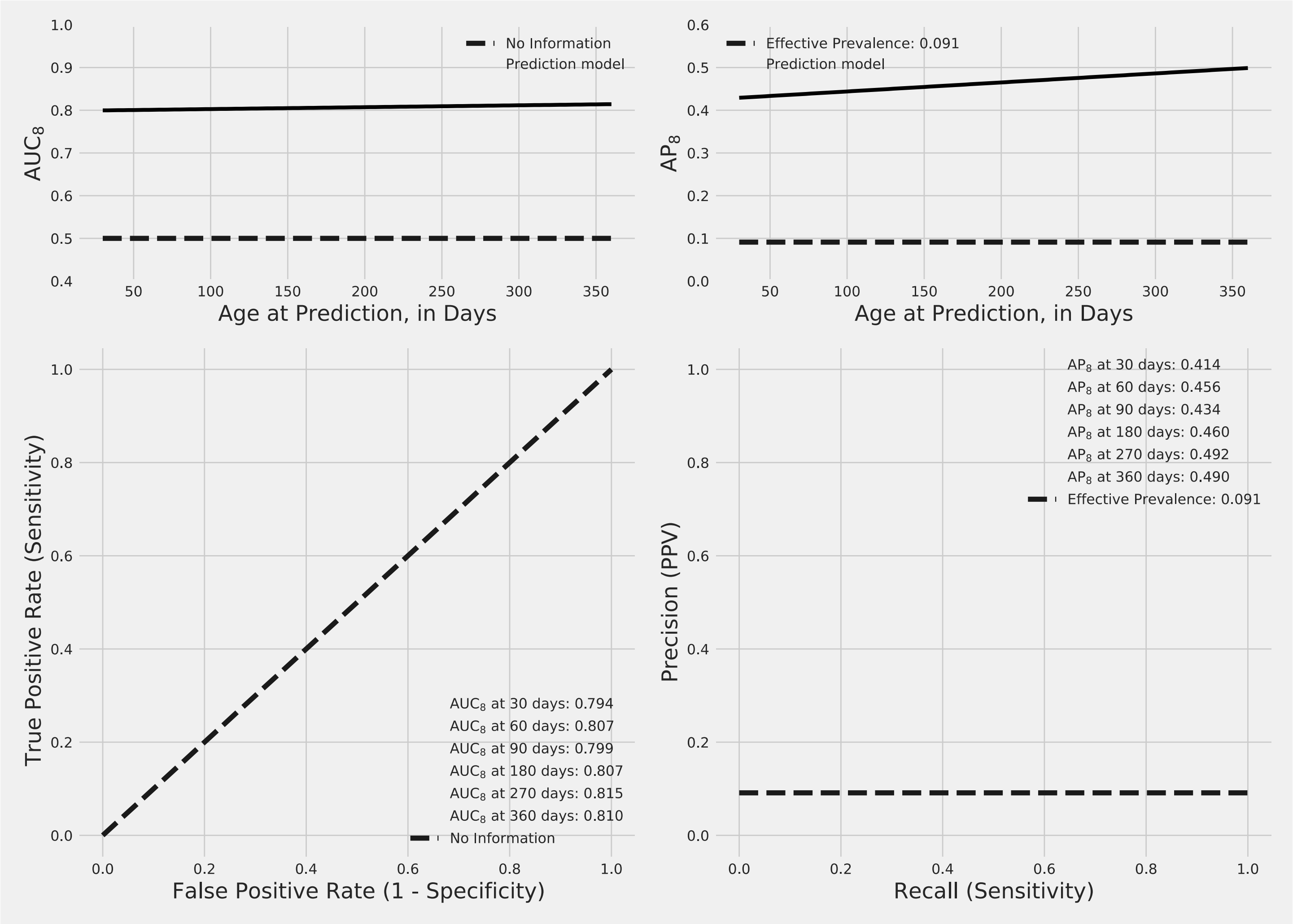

© 2023 Engelhard MM et al. *JAMA Network Open*

eFigure 5. Sensitivity to Follow-up Threshold for 30-Day Models. The panels show the effect of varying the required follow-up threshold *t* from 4 to 10 years when evaluating performance of the 30-days models via the AUC_t and AP_t. In each case are 363 individuals with an observed autism diagnosis in the test set, but the number of controls meeting the required follow-up criterion ranges from 7638 (*t*=4) to 2173 (*t*=10).

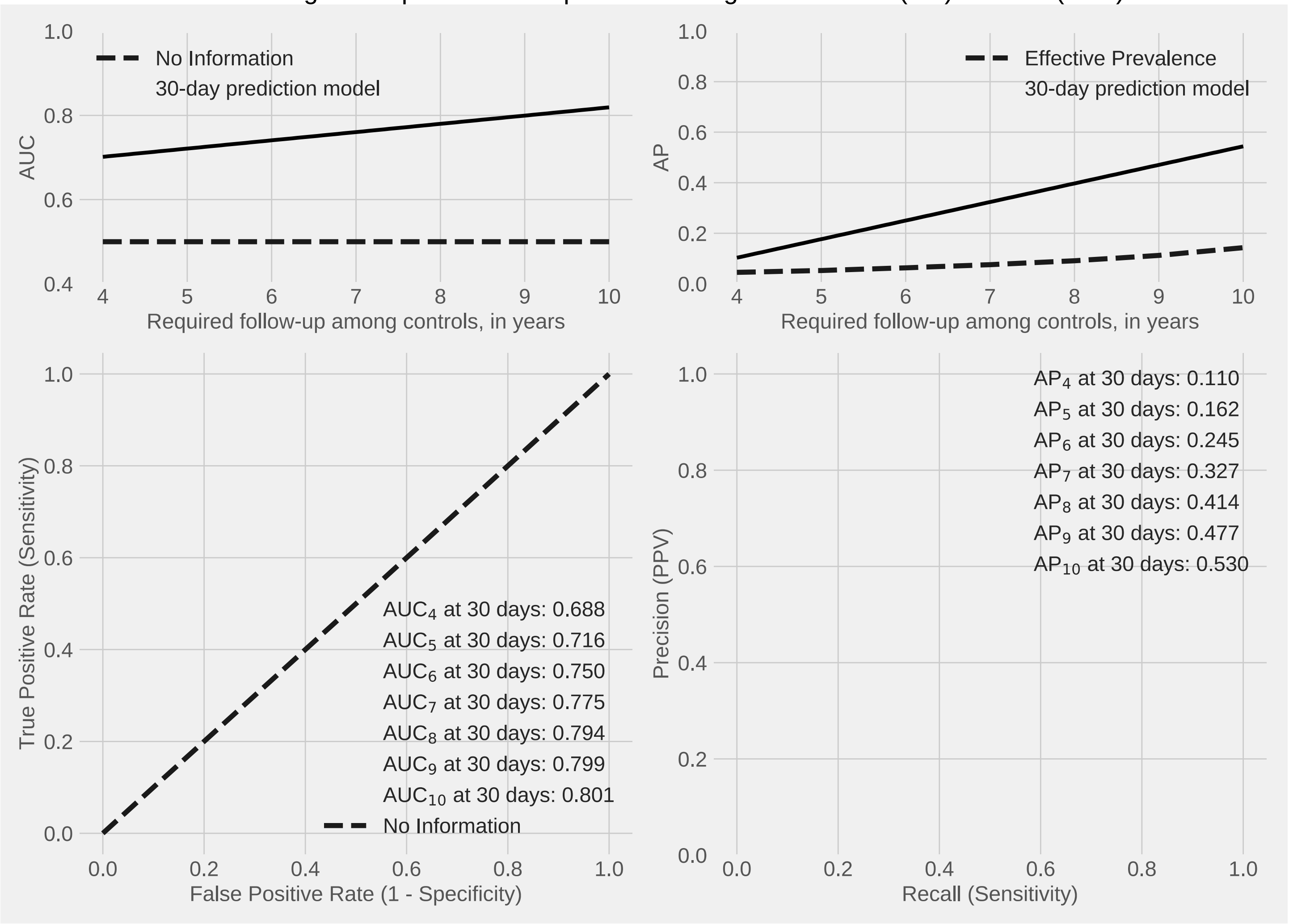

© 2023 Engelhard MM et al. *JAMA Network Open*

eFigure 6. Sensitivity to Follow-up Threshold for 360-Day Models. The panels show the effect of varying the required follow-up threshold *t* from 4 to 10 years when evaluating performance of the 360-days models via the AUC_t and AP_t. In each case are 363 individuals with an observed autism diagnosis in the test set, but the number of controls meeting the required follow-up criterion ranges from 7638 (*t*=4) to 2173 (*t*=10).

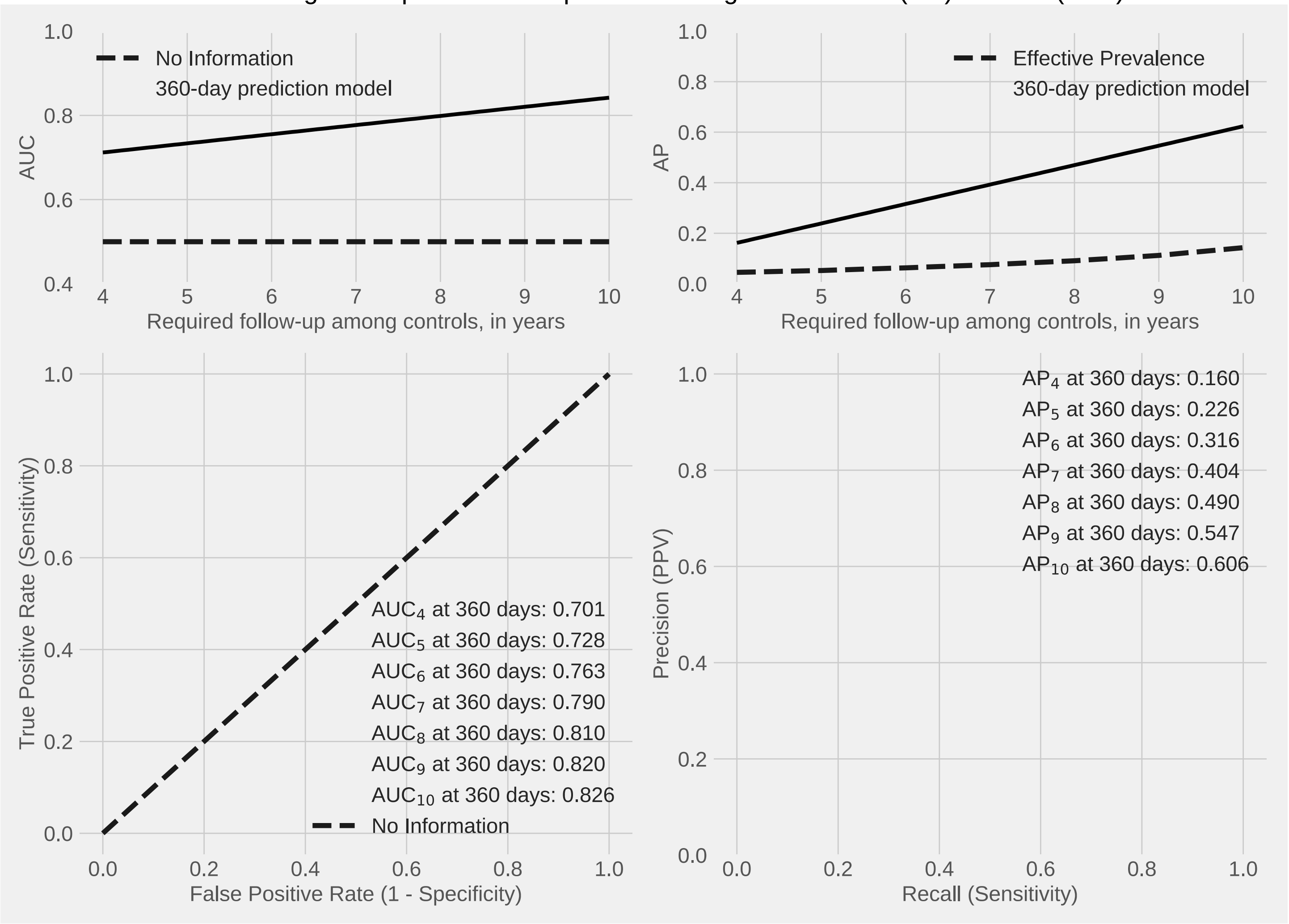

© 2023 Engelhard MM et al. *JAMA Network Open*

eFigure 7. Operating Points. The panels are identical to the bottom two panels of Figure 1 with the addition of markers indicating the position of the high specificity (90%) and very high specificity (97%) operating points referenced in Figure 3 and sTable 2.

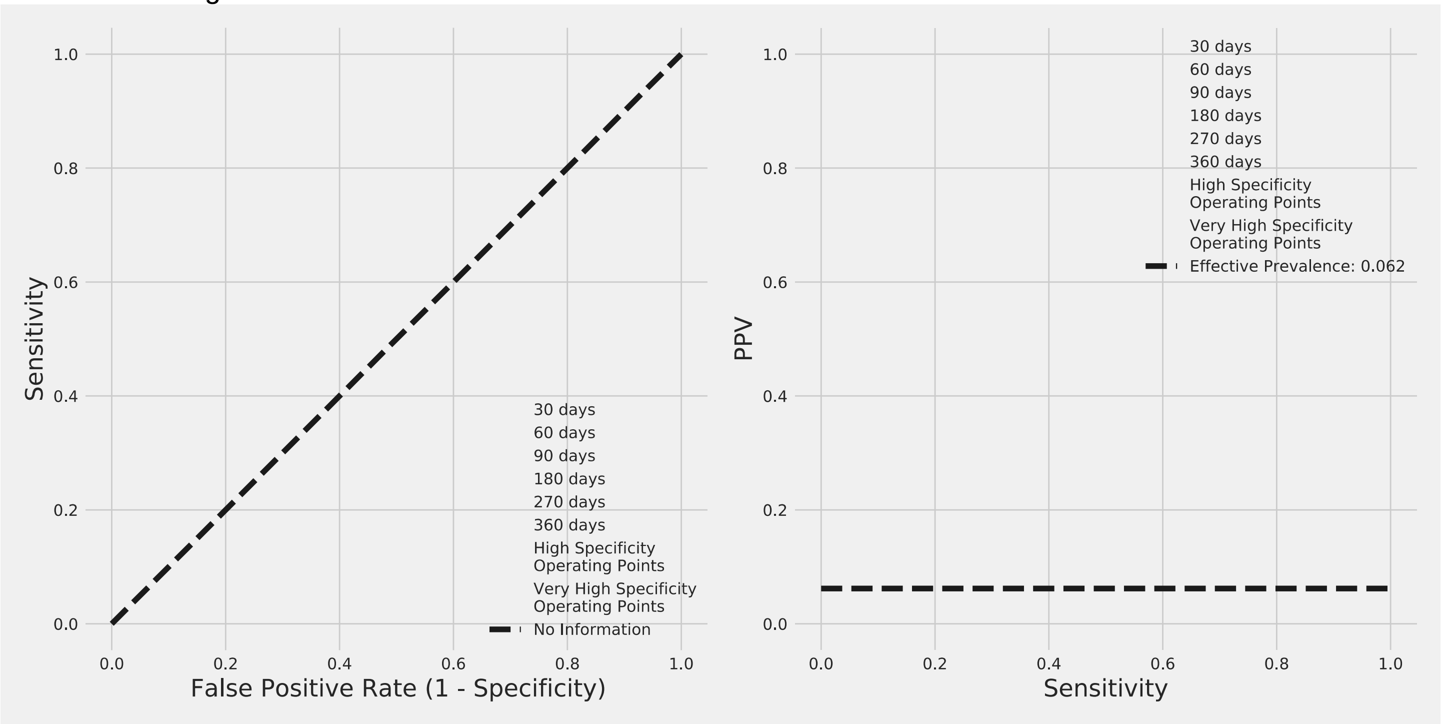

© 2023 Engelhard MM et al. *JAMA Network Open*

eFigure 8. Calibration Curves. Calibration of the 30-day and 360-day prediction models at ages 4 and 8 years. The curves assess correspondence between model-predicted diagnosis probability (e.g. at 4 years) across 8 bins (*i.e.*, risk groups) and the true rates of diagnosis in each bin at that time. The legend shows the Hosmer-Lemeshow calibration statistic (X^2; lower is better) and its associated p-value (higher is better).

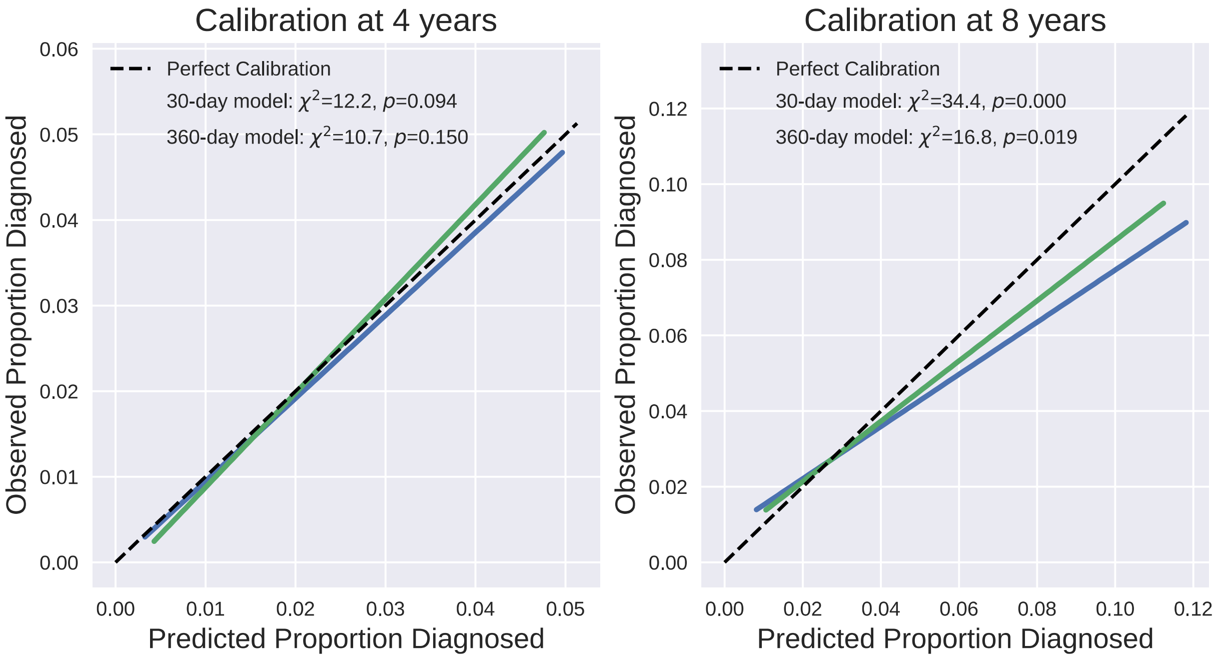

© 2023 Engelhard MM et al. *JAMA Network Open*

eFigure 9. Prediction Performance at 360 Days for Individuals With and Without Other Neurodevelopmental Conditions. This figure is analogous to Figure 2, but shows prediction performance at 360 days rather than at 30 days. Prediction performance is shown for individuals later diagnosed with (a) ADHD, (b) another neurodevelopmental condition, or (c) neither. In each of these groups, cases were defined as children later meeting our autism computable phenotype, and controls were defined as children followed through age 8 but not meeting our phenotype. The top panels summarize the number of cases and controls in each group (top left) and the relationship between autism prevalence and average positive predictive value of model-based prediction (top right). The bottom panels show the tradeoff between sensitivity and specificity (bottom left) and sensitivity and positive predictive value (bottom right) in each group.

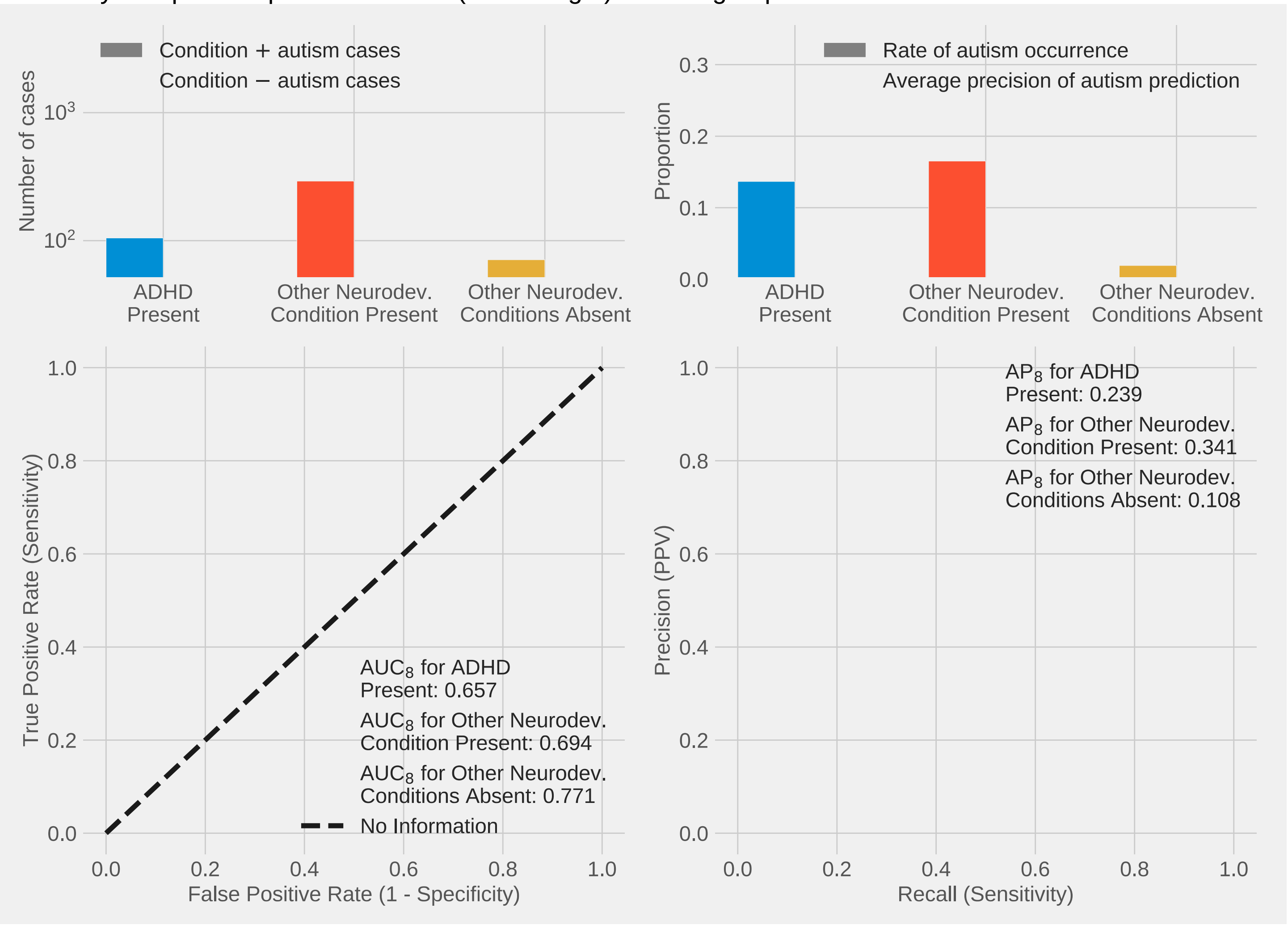

© 2023 Engelhard MM et al. *JAMA Network Open*

eFigure 10. Sensitivity of Prediction Performance Stratified by Race to Follow-up Threshold. The panels show the effect of varying the required follow-up threshold *t* from 4 to 10 years when evaluating differences in performance of the 360-day models between racial groups. The panels show the AUC$_8$ (top), AP$_8$ (middle), and AP$_8$ divided by autism prevalence (bottom) in each group. The dotted lines indicate performance associated with random guessing (*i.e.*, no information).

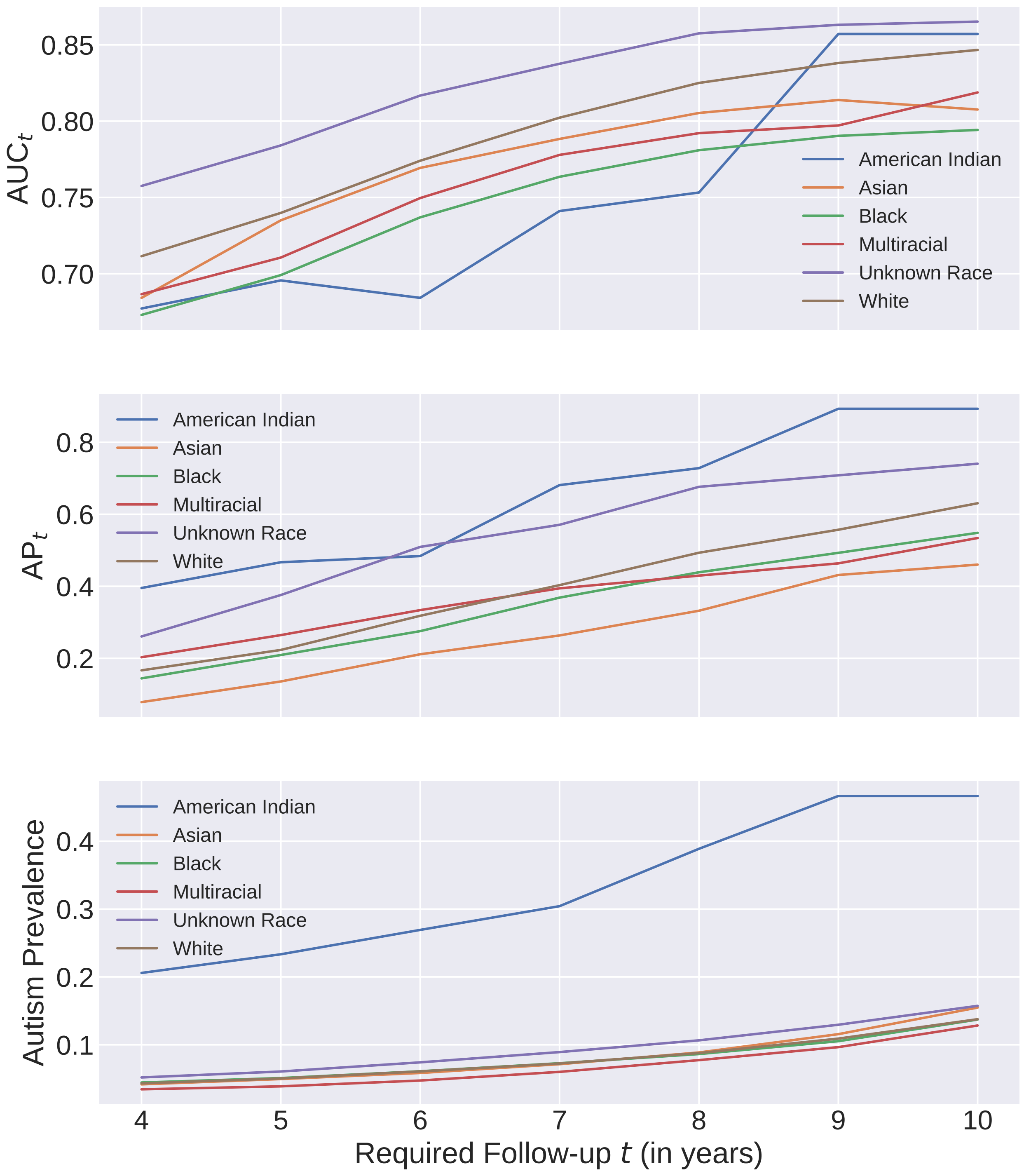

© 2023 Engelhard MM et al. *JAMA Network Open*

eFigure 11. Prediction Performance Stratified by Low Birth Weight. Performance when discriminating between children later diagnosed with autism and children not diagnosed through age 8 among individuals whose earliest recorded weight fell below (True) versus above (False) the 5th percentile based on World Health Organization growth charts. The panels show the AUC_8 (top), AP_8 (middle), and AP_8 divided by autism prevalence (bottom) in each group. The dotted lines indicate performance associated with random guessing (*i.e.*, no information).

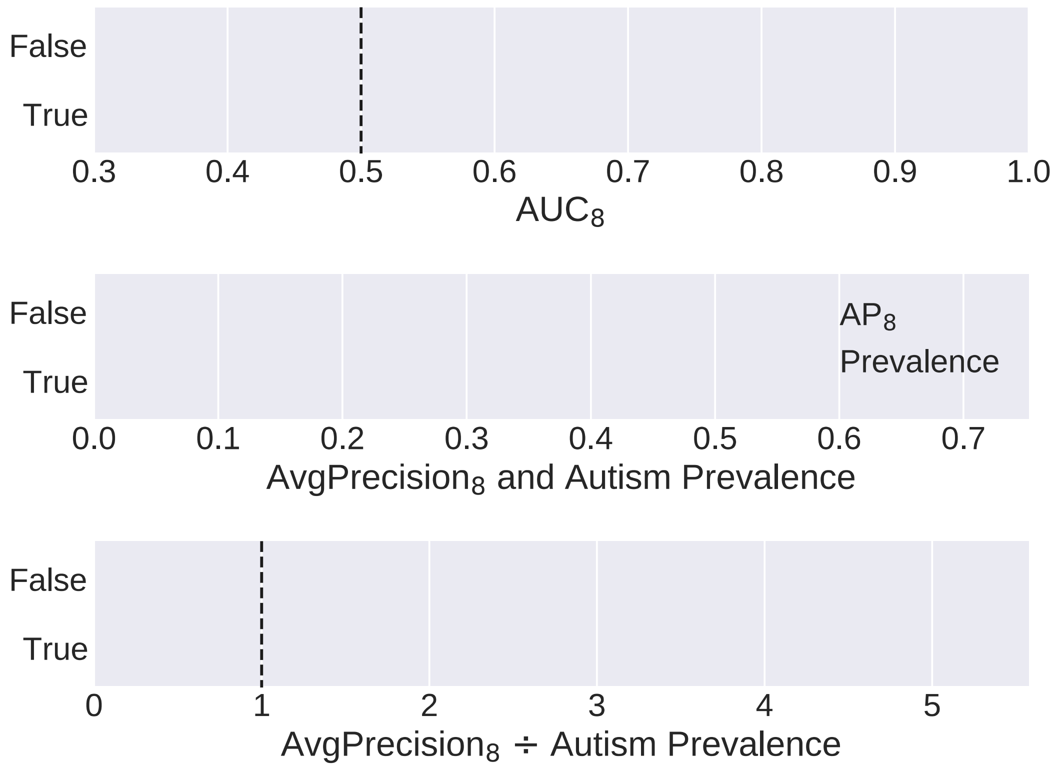

© 2023 Engelhard MM et al. *JAMA Network Open*

eFigure 12. Prediction Performance by Electronic Health Record System (Legacy vs Epic). Performance when discriminating between children later diagnosed with autism and children not diagnosed through age 8 who were born prior to 2013 versus in 2013 or later, whose data was collected after DUHS transitioned to the Epic EHR platform. The panels show the AUC_8 (top), AP_8 (middle), and AP_8 divided by autism prevalence (bottom) in each group. The dotted lines indicate performance associated with random guessing (*i.e.*, no information).

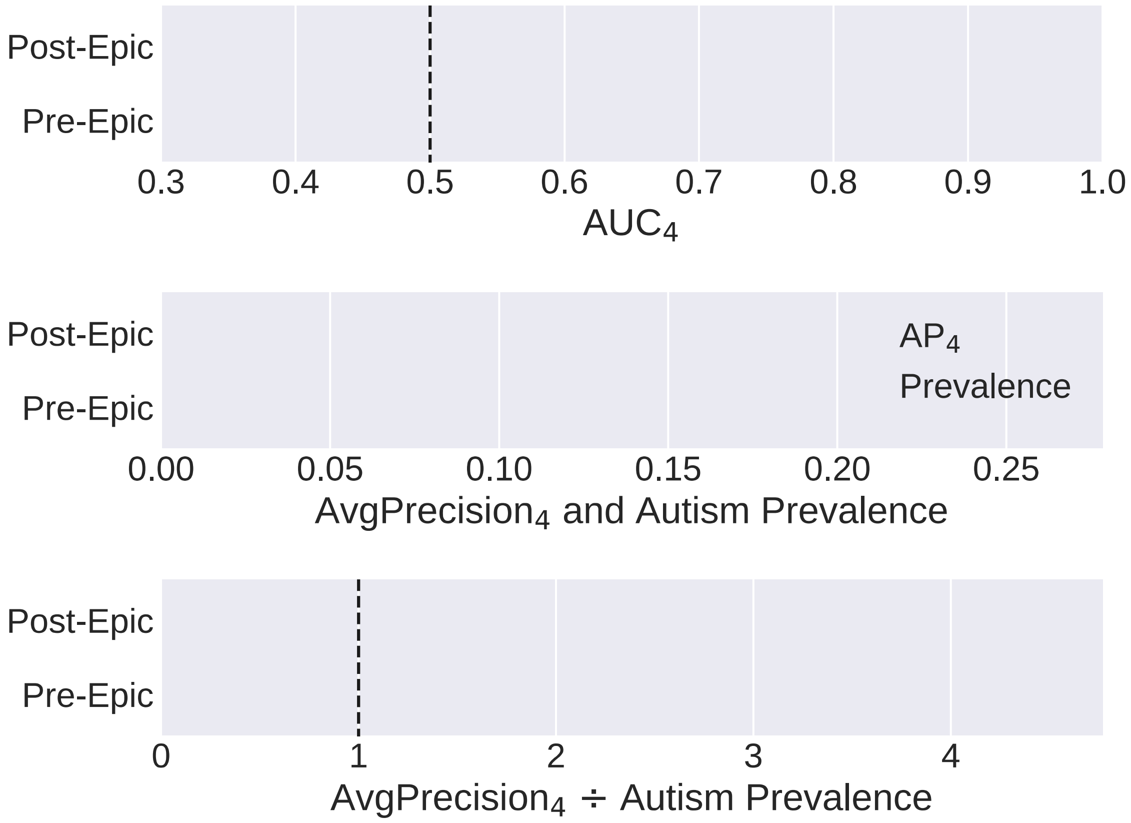

© 2023 Engelhard MM et al. *JAMA Network Open*

eFigure 13. Individual Feature Importance for the 30- and 360-Day Models. The average influence of each predictor on model predictions (i.e., feature importance) for the 30-day (left) and 360-day (right) models was quantified by calculating the average absolute value of all SHAP values for that predictor on individuals in the test set.

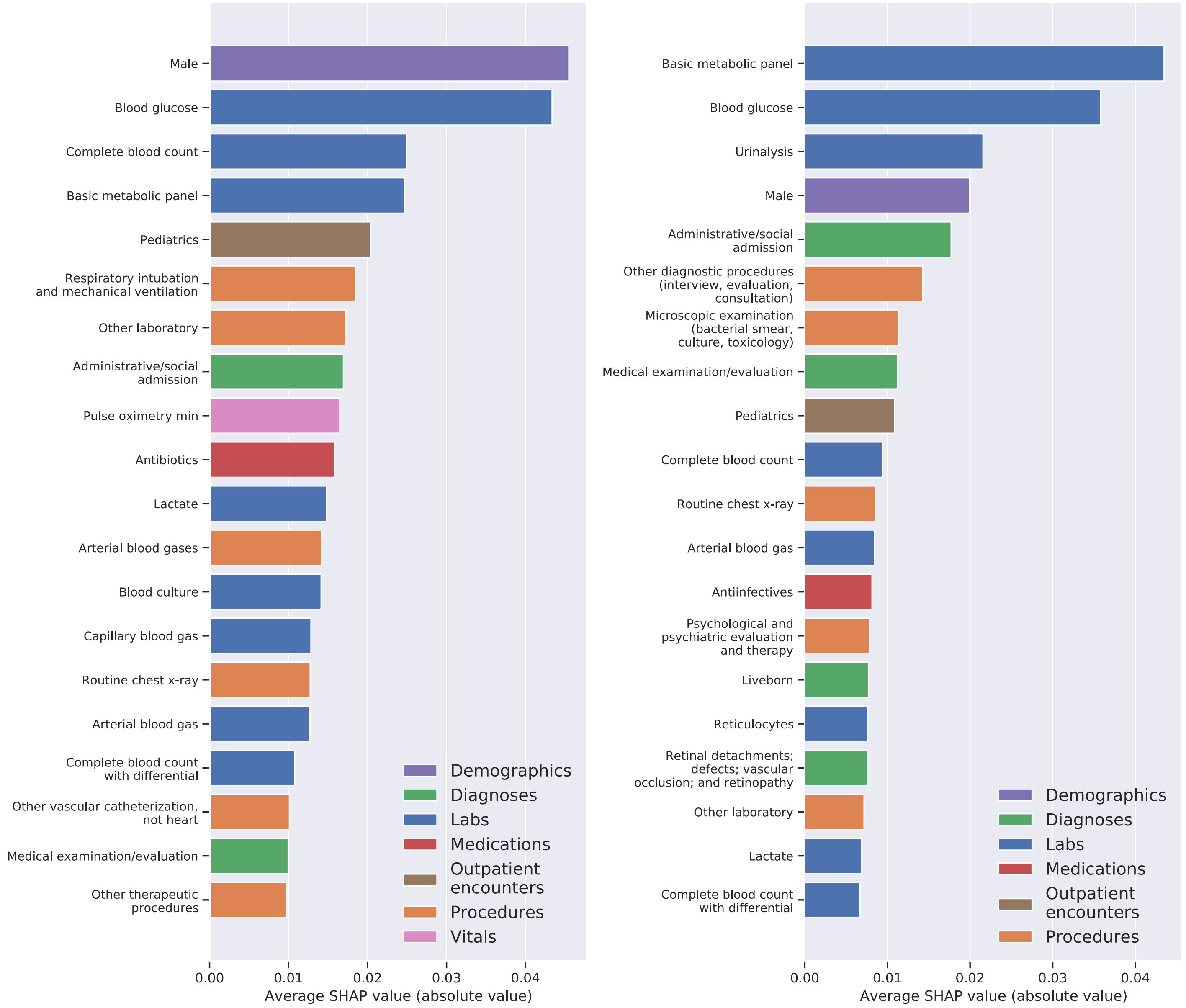

© 2023 Engelhard MM et al. *JAMA Network Open*

eFigure 14. Individual Feature Importance for the 60- and 90-Day Models. The average influence of each predictor on model predictions (i.e., feature importance) for the 60-day (left) and 90-day (right) models was quantified by calculating the average absolute value of all SHAP values for that predictor on individuals in the test set.

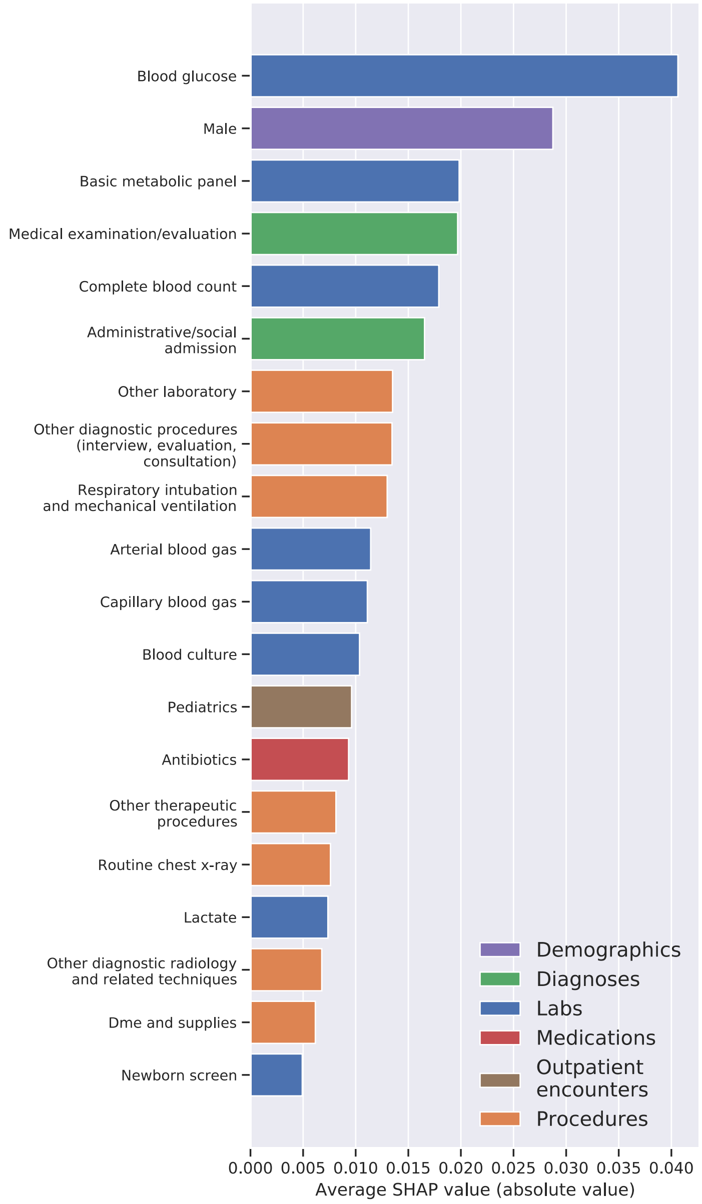

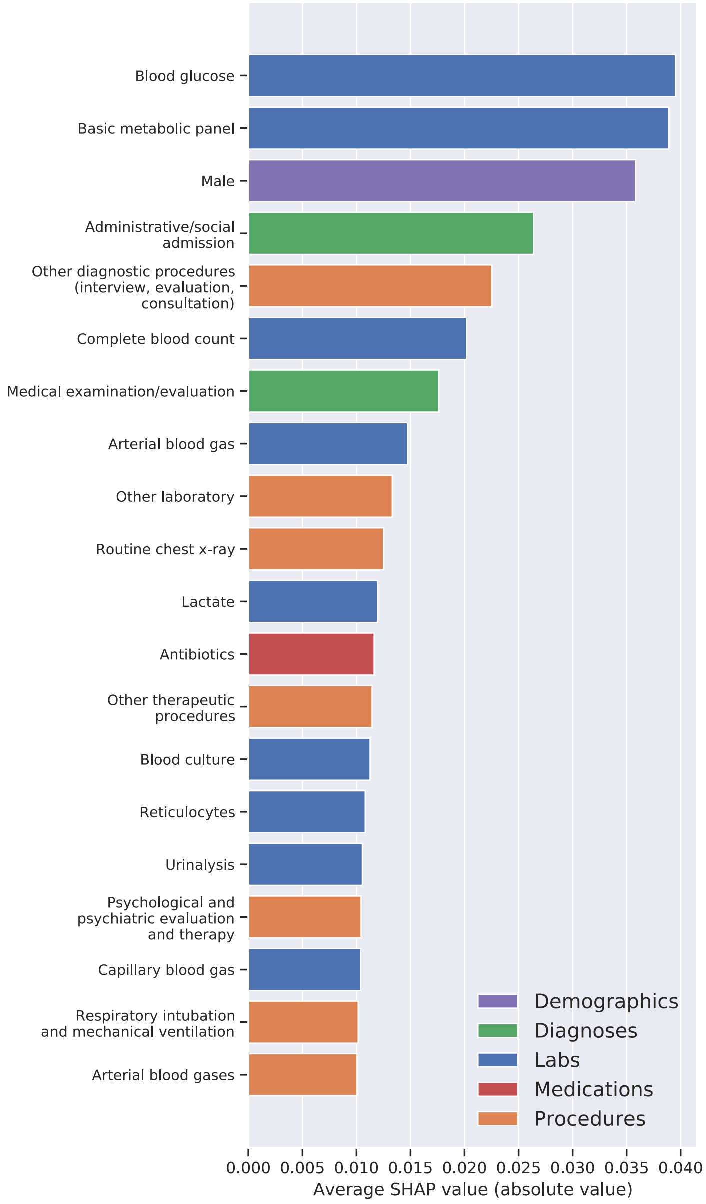

© 2023 Engelhard MM et al. *JAMA Network Open*

eFigure 15. Individual Feature Importance for the 180- and 270-Day Models. The average influence of each predictor on model predictions (i.e., feature importance) for the 180-day (left) and 270-day (right) models was quantified by calculating the average absolute value of all SHAP values for that predictor on individuals in the test set.

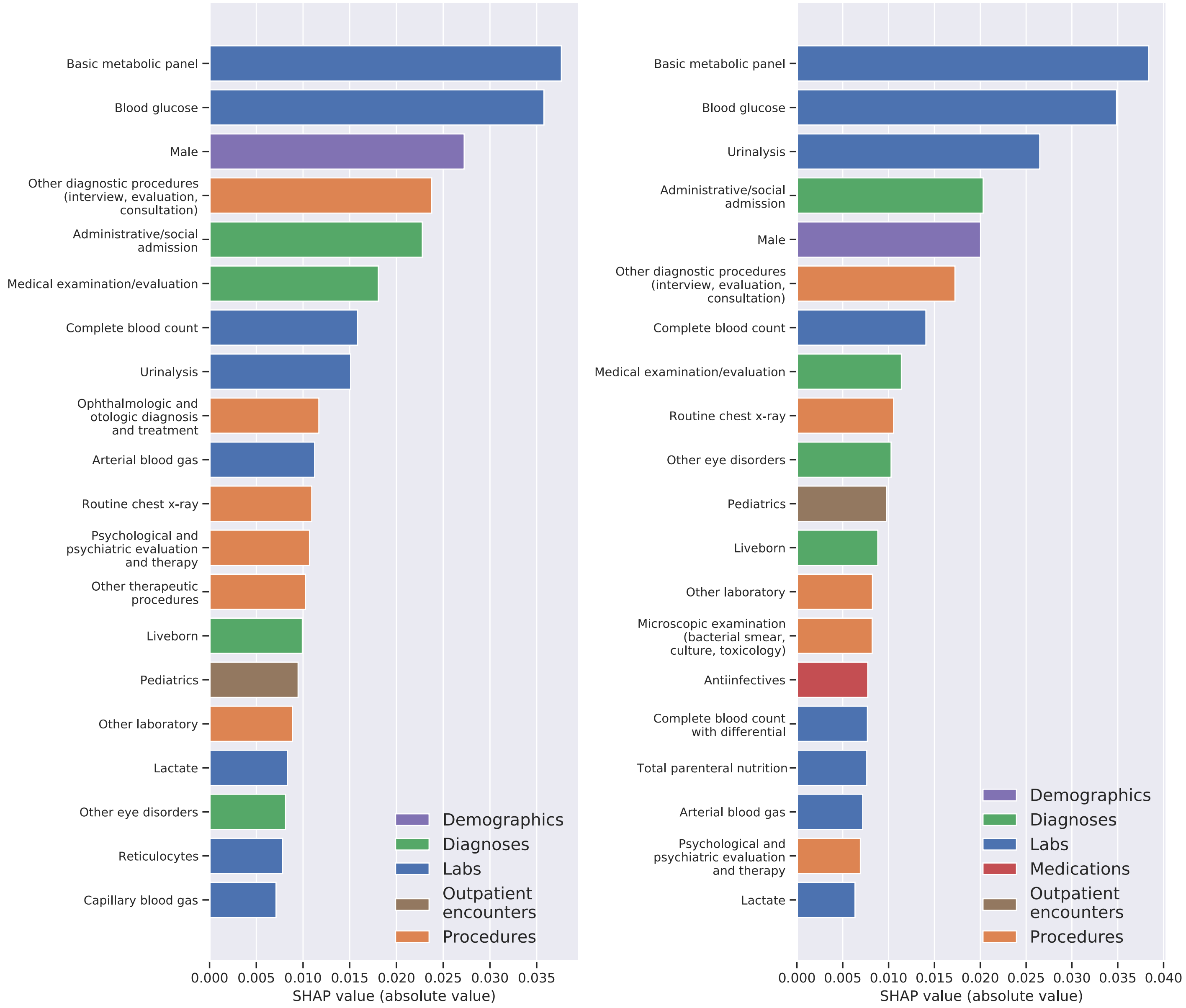

© 2023 Engelhard MM et al. *JAMA Network Open*

eFigure 16. Effect of Training Phenotype. Performance (AUC_8) when discriminating between three groups of individuals: (a) those diagnosed with autism, per our computable phenotype (cases); (b) those with at least one documented autism-related ICD code, but not meeting our phenotype (weak phenotype); and (c) those without a documented autism-related ICD code and followed through at least age 8 (controls). The panels show discrimination of cases versus controls (left), weak phenotype cases versus controls (center), and cases versus weak phenotype cases.

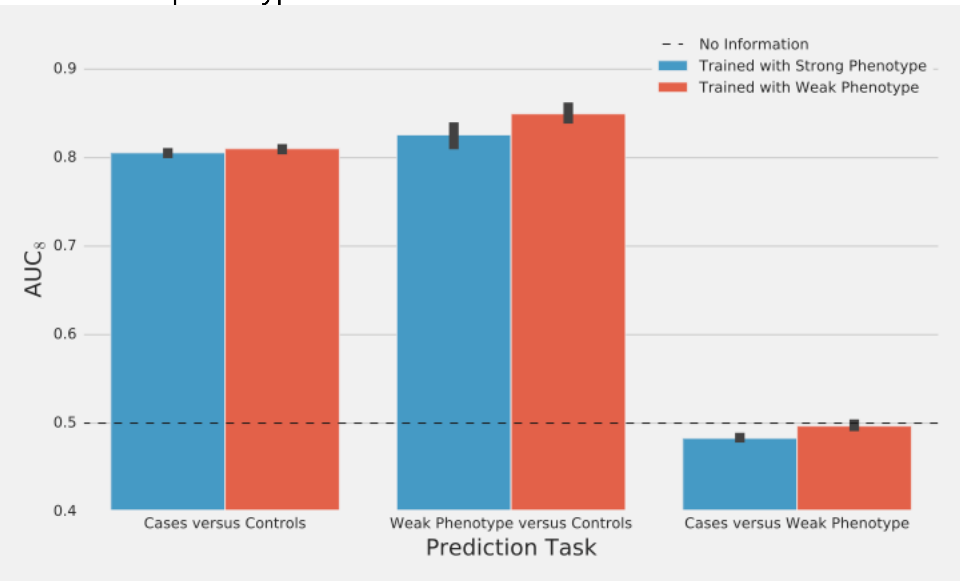

© 2023 Engelhard MM et al. *JAMA Network Open*

eFigure 17. Model-Predicted Risk in the Secondary Evaluation Set. Predictions at 30 days (left panel) and 360 days (right panel) are grouped based on whether they met our computable phenotype for autism (blue versus orange) and whether they were identified by chart review as having been diagnosed (left versus right). Note that the line in both panels (middle left) is a boxplot for the one individual in the test set that was identified by the computable phenotype as having an autism diagnosis but did not meet criteria by chart review.

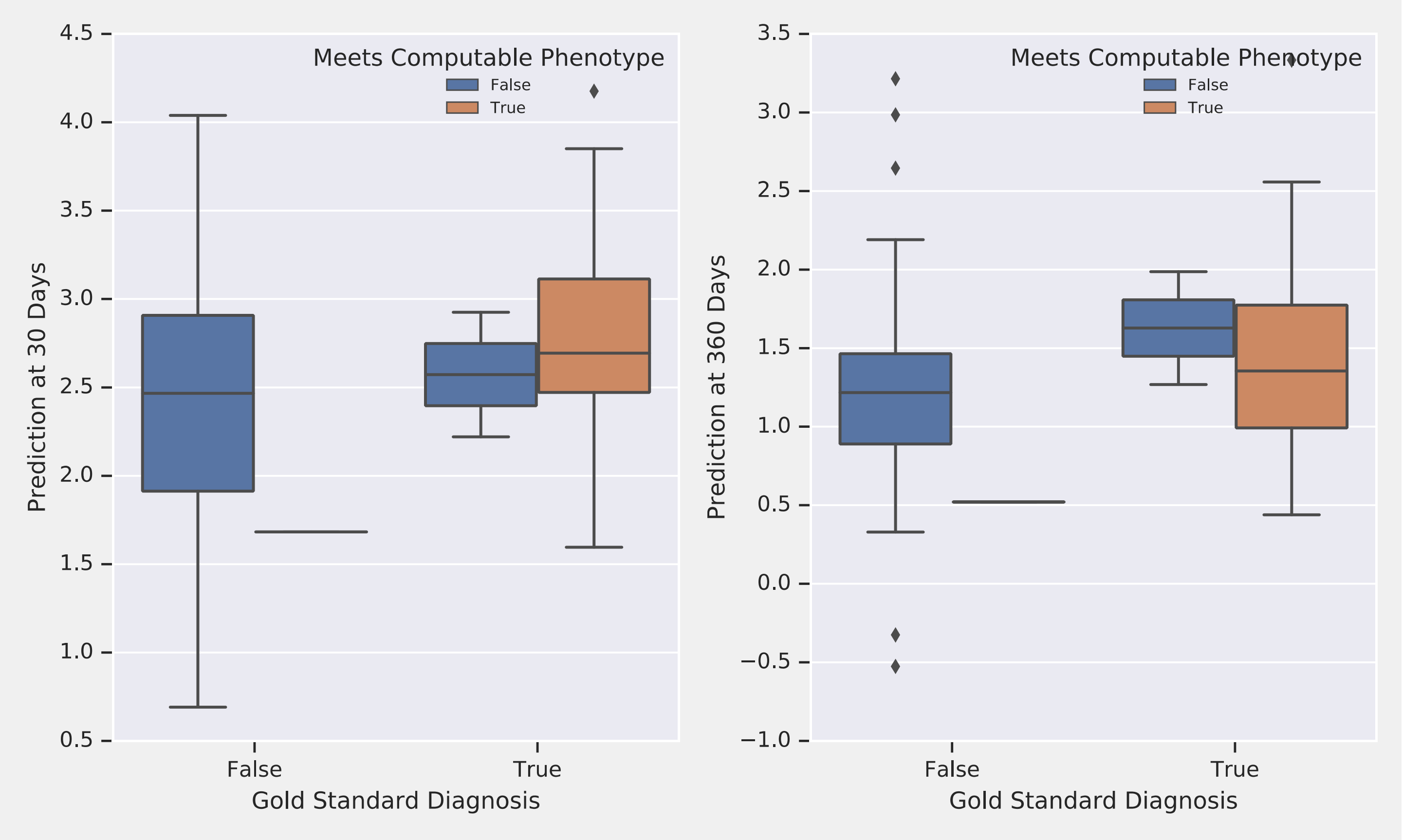

© 2023 Engelhard MM et al. *JAMA Network Open*
