# Supplementary material for: Predictive Value of Early Autism Detection Models Based on Electronic Health Record Data Collected Before Age 1 Year
Source: JAMA Netw Open. 2023 Feb 2;6(2):e2254303. doi: 10.1001/jamanetworkopen.2022.54303 (PMC9896305; doi:10.1001/jamanetworkopen.2022.54303)
Supplement: Supplement 2. — Data Sharing Statement [file jamanetwopen-e2254303-s002.pdf]

Data Sharing Statement

Engelhard. Predictive Value of Early Autism Detection Models Based on Electronic Health Record Data Collected Before Age 1 Year. *JAMA Netw Open*. Published February 02, 2023. doi:10.1001/jamanetworkopen.2022.54303

Data
Data available: No

Additional Information
Explanation for why data not available: This study is based on large-scale electronic health record data from the Duke University Health System, and we do not have permission or the necessary resources to make these data available to others.
